# School-based mindfulness training in early adolescence: what works, for whom and how in the MYRIAD trial?

**DOI:** 10.1136/ebmental-2022-300439

**Published:** 2022-07-07

**Authors:** Jesus Montero-Marin, Matthew Allwood, Susan Ball, Catherine Crane, Katherine De Wilde, Verena Hinze, Benjamin Jones, Liz Lord, Elizabeth Nuthall, Anam Raja, Laura Taylor, Kate Tudor, MYRIAD Team, Sarah-Jayne Blakemore, Sarah Byford, Tim Dalgleish, Tamsin Ford, Mark T Greenberg, Obioha C Ukoumunne, J Mark G Williams, Willem Kuyken

**Affiliations:** 1 Department of Psychiatry, Warneford Hospital, University of Oxford, Oxford, UK; 2 Teaching, Reseach and Innovation Unit, Parc Sanitari Sant Joan de Déu, Sant Boi de Llobregat, Spain; 3 NIHR Applied Research Collaboration (PenARC) South West Peninsula, University of Exeter, Exeter, UK; 4 Department of Psychology, University of Cambridge, Cambridge, UK; 5 UCL Institute of Cognitive Neuroscience, London, UK; 6 King’s College London, King’s Health Economics, Institute of Psychiatry, Psychology and Neuroscience, De Crespigny Park, London, UK; 7 Medical Research Council Cognition and Brain Sciences Unit, University of Cambridge, Cambridge, UK; 8 Department of Psychiatry, University of Cambridge, Cambridge, UK; 9 Department of Human Development and Family Studies, Pennsylvania State University, University Park, Philadelphia, Pennsylvania, USA

**Keywords:** school-based mindfulness training, preventive medicine, mental health, adolescence, process evaluation, moderation, implementation, mediation

## Abstract

**Background:**

Preventing mental health problems in early adolescence is a priority. School-based mindfulness training (SBMT) is an approach with mixed evidence.

**Objectives:**

To explore for whom SBMT does/does not work and what influences outcomes.

**Methods:**

The My Resilience in Adolescence was a parallel-group, cluster randomised controlled trial (K=84 secondary schools; n=8376 students, age: 11–13) recruiting schools that provided standard social–emotional learning. Schools were randomised 1:1 to continue this provision (control/teaching as usual (TAU)), and/or to offer SBMT (‘.b’ (intervention)). Risk of depression, social–emotional–behavioural functioning and well-being were measured at baseline, preintervention, post intervention and 1 year follow-up. Hypothesised moderators, implementation factors and mediators were analysed using mixed effects linear regressions, instrumental variable methods and path analysis.

**Findings:**

SBMT versus TAU resulted in worse scores on risk of depression and well-being in students at risk of mental health problems both at post intervention and 1-year follow-up, but differences were small and not clinically relevant. Higher dose and reach were associated with worse social–emotional–behavioural functioning at postintervention. No implementation factors were associated with outcomes at 1-year follow-up. Pregains−postgains in mindfulness skills and executive function predicted better outcomes at 1-year follow-up, but the SBMT was unsuccessful to teach these skills with clinical relevance.

SBMT as delivered in this trial is not indicated as a universal intervention. Moreover, it may be contraindicated for students with existing/emerging mental health symptoms.

**Clinical implications:**

Universal SBMT is not recommended in this format in early adolescence. Future research should explore social−emotional learning programmes adapted to the unique needs of young people.

**WHAT IS ALREADY KNOWN ON THIS TOPIC**

- There are systematic reviews and meta-analyses demonstrating the potential effectiveness of school-based mindfulness training (SBMT). However, the first arguably adequately powered trial found no main effects, inviting the questions: are there subgroups who do and do not benefit? how does implementation impact effects? and how might SBMT exert any effects?

**WHAT THIS STUDY ADDS**

- This study includes consideration of theoretically driven potential moderators, implementation factors and mediators of a universal SBMT (the ‘.b’ programme). It suggests iatrogenic effects in those with mental health difficulties, and that while mindfulness and executive functioning skills are associated with resilience, this programme does not teach these skills.

**HOW THIS STUDY MIGHT AFFECT RESEARCH, PRACTICE AND/OR POLICY**

- The use of this specific school-based mindfulness curriculum (.b), as a universal intervention for young people in early adolescence, is not indicated. Future research should explore whether different social−emotional trainings might be appropriate to promote mental health, paying close attention to the unique needs of young people in terms of their age and mental health status.

## Background

Mental health problems commonly have their first onset in adolescence, which is a period of heightened vulnerability associated with reduced attentional, emotional and behavioural regulation in the face of growing demands.^1 2^ There is a large body of work developing programmes for adolescents to learn these self-regulation skills as a way to reduce risk of mental ill health and promote well-being.^3 4^ Because of their broad reach and central role in the lives of children and families, schools are seen as the primary setting where such social–emotional learning (SEL) programmes can be provided.^5^

**Figure 1:**
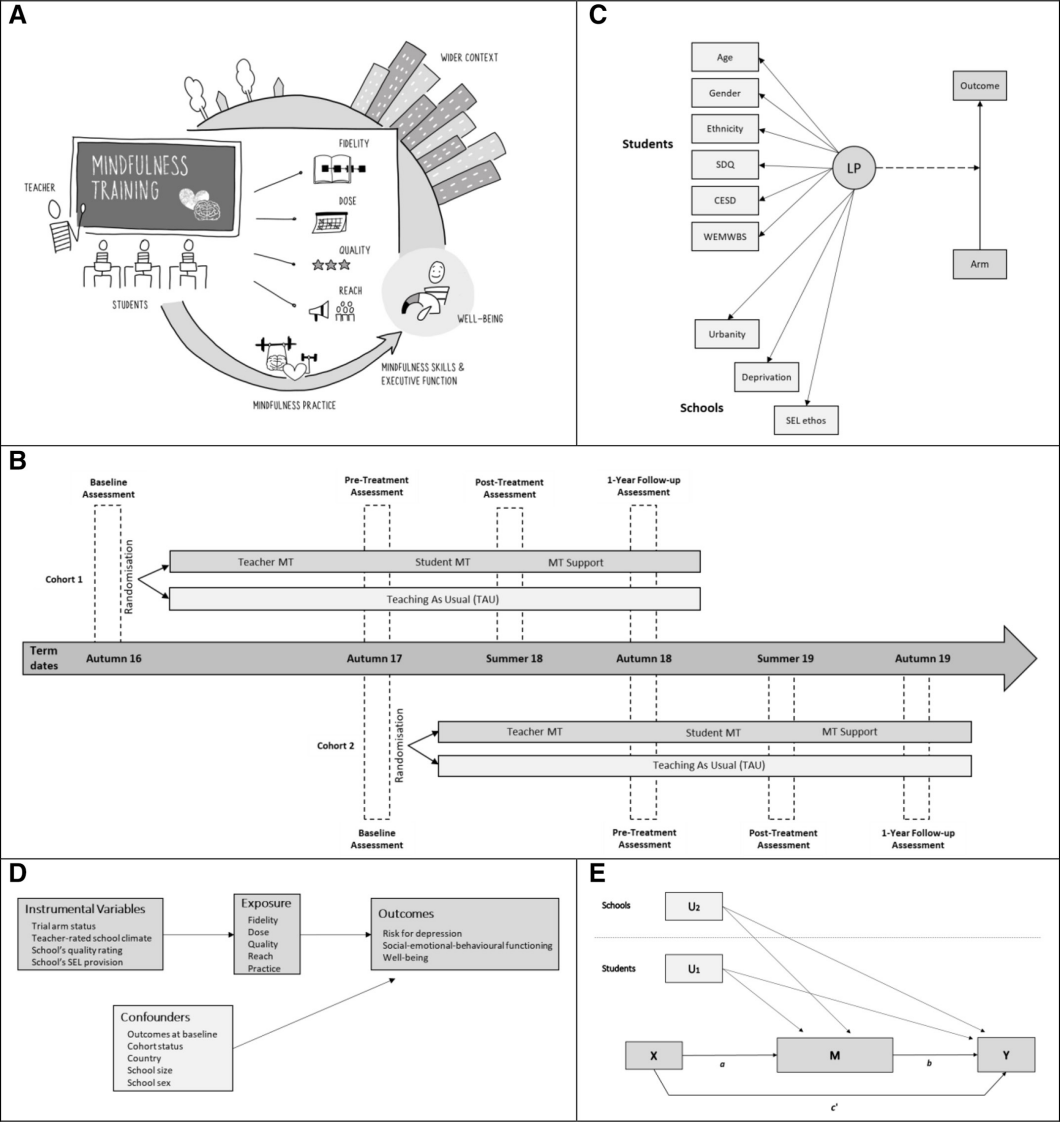
SBMT: what works for whom, how. conceptual framework, design and analytical strategy. (A) Conceptual model for SBMT implementations. Well-being is used here in general to represent outcome variables assessed following implementation of mindfulness training (eg, risk of depression, social–emotional–behavioural functioning and well-being). (B) the MYRIAD trial design. Cohort 1: K=13 schools, cohort 2: K=72 schools. SBMT: K=43 schools; TAU: K=42 schools (1 school allocated to TAU dropped out after randomisation, and the baseline data for pupils from that school were not included in the trial because the school dropped out before the participating classes could be randomly selected for the trial). (C) Mixture model with a secondary auxiliary relationship. The joint model combines the measurement LP hierarchical mixture model and the auxiliary model, where the LP variable is a moderator of a mixed effects linear regression (which accounts for the clustering of observations and adjusts for the student/school-level covariates that are not included in the graph in order to simplify the representation). SDQ: social–emotional–behavioural functioning. CES-D: risk for depression. WEMWBS: well-being. All LP predictors were measured at baseline. (D) Two-stage instrumental variable model to examine the effects of the implementation variables on the primary outcomes, allowing for correlations between observations from the same school. Instruments were entered at the first stage as predictors of implementation. Confounders were introduced at the second stage. (E) Simple mediation path analysis model. The independent variable (X) is the trial arm status. The mediator (M) is (1) the CAMM (mindfulness skills) or (2) the BRIEF-2 (executive function) predifference–post difference, and the dependent variable is the 1-year follow-up measure of the corresponding primary outcome (Y), all measured at the student level. the model accounts for the clustering of observations and adjusts for student-level (U1) and school-level (U2) covariates. The product of *a*×*b* is the indirect effect through the independent variable (X) and mediator (‘I’ or ‘II’), after adjusting for the covariates. c' is the direct effect of the independent variable on the dependent variable after adjustment for the mediating effects and the covariates. BRIEF-2, Behaviour Rating Inventory of Executive Function-2; CAMM, Child–Adolescent Mindfulness Measure; CES-D, Center for Epidemiological Studies for Depression Scale; LP, latent profile; MT, mindfulness training; MYRIAD, My Resilience in Adolescence; SBMT, school-based mindfulness training; SDQ, Strengths and Difficulties Questionnaire; SEL ethos, school social–emotional learning ethos; TAU, teaching as usual; WEMWBS, Warwick-Edinburgh Mental Well-being Scale.

Based on theory, a scoping review and our pilot work,^6^ we developed a high-level conceptual model (figure 1A)^7^ that hypothesises for whom, in what implementation context and how, one such programme (universal school-based mindfulness training (SBMT)) is most likely to be effective. This model sets out potential moderators (eg, the wider school context and characteristics of the schools, teachers, and students), implementation factors (eg, intervention fidelity, dose, quality, reach and mindfulness practice) and mediators (eg, mindfulness skills and executive function).^7^

‘My Resilience in Adolescence’ (MYRIAD) was a cluster randomised controlled trial (RCT) asking if universal SBMT, compared with social–emotional teaching as usual (TAU), promotes mental health and well-being in early adolescence, as implemented in UK secondary schools. In the main analyses, we found no support for the SBMT’s effectiveness compared with TAU on student mental health and well-being outcomes.^8^ There were, however, short-term effects of SBMT versus TAU on teacher burnout and school climate.^9^ This large trial provides an invaluable opportunity to drive innovation and generate hypotheses for future research.

### Objectives

We explored: what works (or is contraindicated) for whom? which implementation factors influence SBMT’s effectiveness? and through what individual-level mediators may SBMT work?

## Methods

We conducted secondary analyses using data from the MYRIAD trial (figure 1B), a superiority two-arm parallel group cluster RCT (ISRCTN86619085; 03/06/2016) that began in 2016 and included 8376 adolescents (age 11–13 years at baseline) in 84 schools (we recruited/randomised 85 schools, but 1 school withdrew post randomisation after baseline) across the UK (see table 1). Recruitment was conducted in two cohorts (recruited in the academic years 2016/2017 and 2017/2018) and involved consenting schools, providing parents with the opportunity to opt their children out and seeking assent from young people themselves. Study design and procedures are presented in full in the study protocol and update.^10 11^ The SBMT and TAU programmes are described in online supplemental A. Consistent with the protocol update,^11^ we used data from the first four time points (baseline, preintervention, post intervention and 1-year follow-up).

10.1136/ebmental-2022-300439.supp1 Supplementary data

**Table 1:** Baseline characteristics of schools and students by trial arm status and overall

School (cluster) characteristics	SBMT	TAU	Total
	**K=43**	**K=41**	**K=84**
Country, n (%)			
England	38 (88)	36 (88)	74 (88)
Scotland, n (%)	2 (5)	1 (2)	3 (4)
Wales	1 (2)	2 (5)	3 (4)
Northern Ireland	2 (5)	2 (5)	4 (5)
Urbanity—urban, n (%)	36 (84)	35 (85)	71 (85)
School size—at least 1000 students, n (%)	20 (47)	22 (54)	42 (50)
Type of school, n (%)			
Mixed	36 (84)	37 (90)	73 (87)
Girls	7 (16)	4 (10)	11 (13)
School quality rating, n (%)			
Requires improvement	6 (14)	5 (12)	11 (13)
Does not require improvement	37 (86)	36 (88)	73 (87)
Deprivation			
% eligible for free school meals, mean (SD)	13.2 (8.1)	11.8 (10.7)	12.5 (9.4)
Provision of PSHE education, mean (SD)	12 (2.5)	12 (2.6)	12 (2.6)
SEL ethos, mean (SD)	50.0 (9.7)	49.9 (10.5)	50.0 (10.1)
	**n=**4232	**n=**4144	**n=**8376
Gender, n (%)			
Female	2350 (56.5)	2159 (53.1)	4509 (54.9)
Male	1724 (41.5)	1823 (44.9)	3547 (43.2)
Age, M (SD)	12.2 (0.6)	12.2 (0.6)	12.2 (0.6)
Ethnicity—white, n (%)	3237 (78.1)	2965 (73.2)	6202 (75.7)
Year group, n (%)			
Year 7	2082 (49.2)	2142 (51.7)	4224 (50.4)
Year 8	1878 (44.4)	1827 (44.1)	3705 (44.2)
Year 9	79 (1.9)	64 (1.5)	143 (1.7)
Year S1, n (%)	193 (4.6)	111 (2.7)	304 (3.6)
Depression (CES-D), mean (SD)	13.6 (10.0)	13.3 (9.8)	13.5 (9.9)
Social–emotional–behavioural functioning (SDQ), mean (SD)	11.8 (6.5)	11.7 (6.4)	11.8 (6.5)
Well-being (WEMWBS), mean (SD)	49.7 (9.7)	49.6 (9.7)	49.7 (9.7)

Data on baseline characteristics were provided for all 43 schools in the intervention arm, and 41 of the 42 schools in the control arm. Sample size ranges from 4157 to 4232 students in the intervention arm and from 4063 to 4144 students in the control arm. In the intervention arm, 4157 students provided data on gender (83 reported other/prefer not to say); 4145 students provided data on ethnicity; 4230 students provided data on CES-D; 4171 on SDQ; and 4214 on WEMWBS. In the control arm, 4063 students provided data on gender (81 reported other/prefer not to say); 4048 students provided data on ethnicity; 4140 students provided data on CES-D, 4081 on SDQ; and 4119 on WEMWBS. School year groups correspond across the home nations as follows: England and Wales, years 7 and 8 (pupils aged 11–12 and 12–13, respectively); Northern Ireland, years 8 and 9 (pupils aged 11–12 and 12–13, respectively); Scotland S1 (pupils aged 12–13). Age and year group data were available for all students. In SDQ, specifically, the Total Difficulties–Self-report scale was used. For further details of the measures, see online supplemental B.

CES-D, Center for Epidemiological Studies for Depression Scale; PSHE, personal, social, health and economic; SDW, Strengths and Difficulties Questionnaire; SEL ethos, school social–emotional learning ethos; WEMWBS, Warwick-Edinburgh Mental Well-Being Scale.

### Measures

Full details of all measures and their references are presented in online supplemental file B.

#### Outcomes

The three primary outcomes (ie, for the main trial) were risk of depression (Center for Epidemiological Studies for Depression Scale (CES-D)), social–emotional-behavioural functioning (Strengths and Difficulties Questionnaire) and well-being (Warwick-Edinburgh Mental Well-being Scale).

#### Moderators

Consistent with our conceptual model and protocol,^7 10 11^ we measured the following potential moderators: baseline age at last birthday (11, 12 and 13 years), gender (male and female), ethnicity (white and other ethnic groups) and the baseline levels of students’ mental health (ie, risk of depression, social–emotional–behavioural functioning and well-being) as student characteristics; urbanicity (rural and urban) as a feature of the schools’ broader context; level of school deprivation (% of students eligible for free school meals) as a characteristic of the school community; and the SEL ethos (a composite measure comprising school quality, teacher-rated school climate and the school’s SEL provision) as a school operational feature.

#### Implementation factors

We assessed the following implementation factors: fidelity (percentage of the original SBMT curriculum covered, from 0% to 100%, evaluated using independent observer ratings of mindfulness teachers trained on the SBMT curriculum); dose (number of SBMT sessions that students attended, ranging from 0 to 10); quality of delivery (using the Mindfulness-Based Interventions Teaching Assessment Criteria, an adapted version for mindfulness training in school settings, ranging from 1=‘incompetent’ to 6=‘advanced’); reach (proportion of study participant students receiving >67% of the SBMT sessions out of the study’s year group population (school level), ranging from 0% to 100%); and the frequency of students’ home-based mindfulness practice during the SBMT period (measured at postintervention) and after the SBMT (measured at 1 year follow-up), using a self-report measure, ranging from 0=‘never’ to 5=‘almost every day’.

#### Mediators

As potential mediators, we considered mindfulness skills, assessed with the ‘Child–Adolescent Mindfulness Measure’, and executive function, measured with the ‘Behaviour Rating Inventory of Executive Function-2’.

### Statistical analyses

This study aimed to analyse potential moderators, implementation factors and mediators of a universal SBMT.

#### Moderator analyses

We explored the aforementioned potential moderators (measured at baseline) of the intervention effect on risk of depression, social–emotional–behavioural functioning and well-being. We used mixed effects linear regression models, allowing for correlations between observations from the same school (clusters), adjusting for cohort status (cohort 1 and cohort 2), country (England, Wales, Scotland and Northern Ireland), school size (large: 1000 children or more, small: fewer than 1000 children), school sex (mixed, female only) and the outcome at baseline. Interaction terms between trial arm status (SBMT vs TAU) and the potential moderators were included in the model to assess moderation. Hedges’ *g* (calculated as the difference in the raw means between trial arms divided by the pooled SD),^12^ as well as the adjusted mean difference (AMD), were provided, together with their corresponding 95% CIs. We fitted separate models for each moderator and each primary outcome at 1-year follow-up; we also present results at postintervention.

We augmented the traditional moderation framework by examining different types of students defined by their individual/contextual characteristics. For that, we carried out a latent profile analysis (LPA) that was developed in three steps and included the following manifest variables: student characteristics (age, gender, ethnicity, risk of depression, social–emotional–behavioural functioning and well-being), and the school’s broader context (school urbanity), community (school deprivation) and operational features (school social–emotional learning ethos (SEL ethos)). For more details on the LPA, see online supplemental C. We fitted a joint model that combined the measurement latent profile (LP) mixture model and the auxiliary model where the LP was a moderator of a mixed effects linear regression (figure 1C). Models allowed for correlations between observations from the same school (cluster) and adjusted for the covariates mentioned previously.

#### Implementation analyses

Instrumental variable methods were used to examine the effects of the implementation factors (dose, quality, reach, fidelity and home-based mindfulness practice) on the three primary outcomes, accounting for hidden confounding relationships between the implementation factors and the outcomes. A two-stage least squares instrumental variable approach,^13^ allowing for correlation between observations from the same school (cluster), was used to examine the effects of the implementation variables on the three primary outcomes at postintervention and 1-year follow-up. Teacher-rated school climate, the school’s quality rating and the school’s SEL provision (online supplemental B) were independently assessed and entered at the first stage as predictors of implementation. Adjustments for cohort status, region, school size, school sex and the outcome at baseline were introduced at the second stage as predictors of the outcome. Trial arm status was used as the instrumental variable for each implementation factor (figure 1D). Unstandardised regression coefficients, 95% CIs and p values are reported.

#### Mediation analyses

We explored the potential indirect relationship between trial arm status (independent variable) and primary outcomes at 1-year follow-up (dependent variables), through pregains–postgains in (1) mindfulness skills, and (ii) executive function (process measures), on the theoretical premise that mindfulness training aims to improve both mental processes, which in turn might improve mental health.^14 15^ We used a simple mediation path analysis model, allowing for correlations between observations from the same school (cluster), adjusting for the outcome at baseline, cohort status, country, school size and school sex. We calculated p values for each path coefficient using the delta method, and the 95% CI for the indirect effect (IE) (ie, based on a Monte Carlo simulation).^16^ For that, we estimated the joint distribution of the path coefficients (figure 1e) using 20 000 random draws from the parameter estimates and their associated asymptotic variances/covariance. IEs are considered significant when their 95% CIs do not include zero. The proportion of the variation in the mediator that is explained by trial arm status and the proportion of the variation in the outcome that is explained by the mediator were calculated.^17^ Model fit was quantified using both the comparative fit index and the root mean square error of approximation.

The analyses followed a complete case approach (as can be seen in online supplemental tables S2–S4), low proportions of missing data and minimal sociodemographic differences between students with/without missing data were observed).^18^ The significance level was set at 0.05 using two-sided tests. Given the exploratory nature of this study, we did not correct for multiple testing. Analyses were performed using Stata V.17.0, Mplus V.8.7, and R v4.0.5.

## Findings

Of the students recruited to the trial, 91% (89% in SBMT and 92% in TAU) provided data on at least one primary outcome at postintervention, and 87% (87% in SBMT and 86% in TAU) provided data at 1-year follow-up (online supplemental tables S1 and S2). Student baseline characteristics by postintervention and 1-year follow-up status are presented in online supplemental tables S3 and S4.

### Moderation

Descriptive data for all potential moderators at baseline can be found in online supplemental table S1. At baseline, students had a mean (SD) age of 12.2 (0.6) years, 54% identified as female and 74% as ‘white’. As illustrated in online supplemental B, most students were in the ‘low’ risk category of risk for depression (67.3%), and in the ‘normal’ risk category for social–emotional–behavioural functioning (71.5%). The mean scores on the primary outcomes at baseline indicate that we recruited a nationally representative sample of students regarding risk for depression (mean=13.5, SD 9.9)^19^ and well-being (mean=49.7, SD 10.0),^20^ with slightly poorer social–emotional–behavioural functioning (mean=11.8, SD 6.5).^21^ The broader school context was mainly ‘urban’ (84.5%). The mean percentage of students eligible for free school meals per school was 12.5% (SD 9.4%), and 36% of schools had a higher percentage of students eligible for free school meals than the national median (SD 29.4%). The school’s SEL ethos had a mean value of 50.0 (SD=10.1, range=0 to 100), meaning that SEL ethos was in the medium range. Sample characteristics were highly similar between the trial arms (table 1).

As can be seen in online supplemental tables S5–S10, age showed a trend moderating the intervention effect on risk of depression at postintervention (p value for the interaction=0.052), with SBMT, relative to TAU, resulting in higher risk of depression in the youngest students (AMD=0.91, 95% CI 0.07 to 1.76).

Online supplemental E presents the model selection, latent moderator interpretation and student classification according to the LPA to explore the impact of risk for mental health problems on outcomes by trial arm. A two-level LP model—LP I: ‘low risk’ for mental health problems; LP II: ‘high risk’ for mental health problems—was estimated. Online supplemental figure S1 includes a graphical representation of the distribution of predictor variables between LPs (online supplemental table S11) shows the LPs and their associated baseline characteristics by trial arm. The LPs moderated the intervention effect on risk of depression at postintervention (p value for the interaction=0.016) and 1-year follow-up (p value for the interaction=0.023), as well as on well-being at postintervention (p value for the interaction=0.050) and 1-year follow-up (p value for the interaction=0.029). Students in the ‘high-risk’ LP who received SBMT, compared with those that were in the ‘high-risk’ LP but received TAU, reported significant detrimental effects on risk of depression (postintervention: AMD=1.40, 95% CI 0.27 to 2.53; 1-year follow-up: AMD=1.47, 95% CI 0.37 to 2.57, and well-being (post-intervention: AMD=−1.10, 95% CI −1.98 to −0.22; 1-year follow-up: AMD=−0.88, 95% CI −1.71 to −0.05) (online supplemental figure S2 and table S12).

### Implementation

Online supplemental table S13 presents descriptive data for all implementation factors. The mean number of SBMT sessions attended was 9.0 (SD 2.1), out of 10. Mean fidelity to the SBMT programme was 83.0% (SD 12.1) of the original content being taught. Mean quality in delivering the SBMT was 3.8 (SD0.8) out of 6. Mean reach was 25.7% (SD 11.4) of students in the study’s year group school population receiving more than 67% of the SBMT sessions. The mean frequency of students’ home-based mindfulness practice was 1.2 (SD 1.1, median 1.0, IQR 0.2–1.8) during the SBMT and 0.8 (SD 0.9, median 0.5, IQR 0.0–1.3) after the SBMT, with possible range from 0 to 5.

None of the implementation factors were significantly related to any of the primary outcomes at 1-year follow-up (table 2 and online supplemental table S14). At postintervention, higher dose was related to detrimental effects on social–emotional–behavioural functioning and well-being, and higher reach with worse social–emotional–behavioural functioning, all with small effects (online supplemental tables S15 and S16).

**Table 2:** Instrumental variable analysis of primary outcomes at 1-year follow-up, with allocated group as an instrument for the implementation variables

Outcome/implementation variables	N (K)	Coefficient	95% CI	P value
Risk of depression				
Dose	5508 (65)	0.08	−0.01 to 0.17	0.065
Fidelity	5667 (66)	0.01	−0.004 to 0.02	0.219
Reach	5673 (65)	0.02	−0.01 to 0.04	0.284
Quality	6139 (73)	0.12	−0.07 to 0.31	0.199
Practice (postintervention)	5960 (73)	0.40	−0.24 to 1.03	0.226
Practice (1 year follow-up)	6196 (73)	0.56	−0.33 to 1.44	0.218
Social–emotional–behavioural function				
Dose	5423 (65)	0.05	−0.001 to 0.09	0.056
Fidelity	5577 (66)	0.01	−0.001 to 0.01	0.109
Reach	5580 (65)	0.01	−0.01 to 0.02	0.185
Quality	6046 (73)	0.07	−0.03 to 0.58	0.150
Practice (postintervention)	5874 (73)	0.18	−0.14 to 0.49	0.274
Practice (1-year follow-up)	6106 (73)	0.26	−0.18 to 0.71	0.243
Well-being				
Dose	5484 (65)	−0.03	−0.11 to 0.04	0.363
Fidelity	5643 (66)	−0.001	−0.01 to 0.01	0.781
Reach	5649 (65)	−0.004	−0.03 to 0.02	0.742
Quality	6116 (73)	−0.02	−0.18 to 0.15	0.849
Practice (postintervention)	5936 (73)	−0.003	−0.55 to 0.54	0.992
Practice (1-year follow-up)	6168 (73)	−0.02	−0.76 to 0.73	0.967

Coefficient: unstandardised regression coefficient (slope) of the instrumental variable analysis (representing the increase in the outcome per 1 unit increase in the predictor) with cluster-robust maximum likelihood estimation, including schools (clusters) as random effects, and adjusted for the baseline levels of student mental health (ie, risk of depression, social–emotional–behavioural functioning, well-being), cohort, school size, school sex, and country. Dose is the number of SBMT sessions that students received. Quality is the teacher competency delivering the SBMT independently evaluated by using the Mindfulness-based Interventions Teaching Assessment Criteria. Fidelity is the independently rated percentage of the total original SBMT content delivered by the teacher. Reach is the percentage of school study students receiving ≥67% of SBMT sessions. Practice is the amount of home-based student mindfulness practice during/after the intervention.

K, number of clusters (schools) in analysis; n, number of students in analysis; p, p value for the slope; SBMT, school-based mindfulness training.

### Mediation

At preintervention, students had a mean score of 27.6 (SD 7.9) on mindfulness skills (possible range=0–40) and of 83.7 (SD 20.8) on executive function (possible range=52–156). Mindfulness skills and executive function were similar between the SBMT and TAU arms at each time point (online supplemental table S17).

As shown in table 3, being randomised to SBMT versus TAU produced significant IEs—the 95% CIs excluded zero—on risk of depression at 1-year follow-up, through pregains–postgains in mindfulness skills (unstandardised 95% CI −0.010 to −0.0001); and on social-emotional-behavioural functioning at 1-year follow-up, through pregains–postgains in executive function (unstandardised 95% CI −0.015 to −0.006). In general, preintervention‒postintervention improvements in mindfulness skills and executive function were significantly related to 1-year follow-up scores in the three primary outcomes, with small-to-medium effects. However, in the mentioned models, being randomised to SBMT versus TAU induced very small (although statistically significant) preintervention‒postintervention improvements in mindfulness skills and executive function, with no clinically important relevance.

**Table 3:** Path estimates and IEs of trial status through mindfulness skills or executive function on the primary outcomes of risk for depression, social–emotional–behavioural functioning, and well-being

Outcome/mediator	N	A (SE)	P	R^2^ (a)	B (SE)	P	R^2^ (b)	c’ (SE)	P	IE	MC 95% CI	CFI	RMSEA
Risk of depression													
Mindfulness skills	7865	0.02 (0.01)	0.050	0.001	−0.28 (0.02)	<0.001	0.23	0.06 (0.32)	0.844	−0.01	−0.010 to −0.001	0.971	0.029
Executive function	7683	−0.02 (0.02)	0.388	0.000	0.13 (0.01)	<0.001	0.23	0.25 (0.33)	0.440	−0.003	−0.008 to 0.003	0.994	0.014
Social–emotional–behavioural function													
Mindfulness skills	7755	0.03 (0.02)	0.055	0.001	−0.15 (0.01)	<0.001	0.31	0.09 (0.16)	0.555	−0.004	−0.008 to 0.000	0.979	0.030
Executive function	7575	−0.12 (0.03)	<0.001	0.002	0.09 (0.01)	<0.001	0.33	0.19 (0.15)	0.226	−0.01	−0.015 to −0.006	0.997	0.012
Well-being													
Mindfulness skills	7835	−0.01 (0.01)	0.380	0.000	0.17 (0.02)	<0.001	0.19	0.16 (0.27)	0.554	−0.002	−0.004 to 0.001	0.965	0.029
Executive function	7654	0.01 (0.02)	0.818	0.000	−0.09 (0.01)	<0.001	0.20	0.05 (0.28)	0.871	0.000	−0.004 to 0.003	0.991	0.015

N is the number of students (number of clusters (schools)=84). R^2^ (a) is the proportion of the variation in the mediator that is accounted for by the independent variable. R^2^ (b) is the proportion of the variation in the dependent variable that is accounted for by the mediator. a is the unstandardised path ‘a’ (see figure 1E); b is the unstandardised path ‘b’ (see figure 1E); c’ is the unstandardised direct effects after controlling for the mediator and covariates (see figure 1E). IE is the unstandardised maximum likelihood robust-based regression coefficient for the mediational models, reflecting the IE (a×b) of group allocation on follow-up scores in the primary outcomes of risk of depression, social–emotional–behavioural functioning, and well-being (controlling for the baseline levels of the primary outcomes, cohort, country, school size, school sex, and adjusted for the clustering of observations) through prechanges to postchanges in the process outcomes of self-reported mindfulness skills or executive function. MC 95% CI is the 95% confidence interval for the IE based on a Monte Carlo simulation of the joint distribution of the corresponding slopes using 20000 random draws from the parameter estimates and their associated asymptotic variances and covariance.

CFI, comparative fit index; IE, indirect effect; RMSEA, root mean square error of approximation.

## Discussion

The MYRIAD trial premises were that there is an urgent need to prioritise mental health in early adolescence, as early/mid-adolescence is a developmental window when many mental health problems emerge; and schools may play an important role in fostering mental health by teaching foundational abilities such as mindfulness skills and executive function. As reported elsewhere,^9^ there was no effect on any of the primary outcomes of SBMT versus TAU in students. The present study explored for whom SBMT does/does not work, what implementation context influences SBMT’s effectiveness, and how SBMT works. Our ultimate aim is to inform innovation and research on the prevention of mental health problems in early adolescence.

We used a universal SBMT (‘.b’) that comprised psychoeducation, using mainstream educational methods and very brief mindfulness practices delivered by schoolteachers who had undergone bespoke training. Possibly a more engaging format, curriculum with a different focus (eg, key mechanisms of risk/resilience), pedagogical approach (eg, facilitating the acquisition of these skills), length of the curriculum (eg, shorter but more frequent sessions) or mode of delivery (eg, by more highly trained teachers) may have been more accessible, engaging and effective. A recent study observed that expert facilitators teaching a multi-component SEL curriculum in a full-time basis may be effective.^22^ Schoolteachers can benefit from mindfulness training,^23^ and this can potentially benefit students through improvements in teacher well-being, classroom instruction, and school climate.^9^ In the absence of compelling evidence, our results do not support the universal roll-out of SBMT, at least in the form of this curriculum.

Our data suggest that adolescents at different developmental stages may require benefit from different approaches. Adolescence is a period of significant social–cognitive–emotional development. Younger adolescents (age 11) may have more limited ability to learn and apply mindfulness skills than somewhat older teens. This is because the skills taught in this curriculum require substantial metacognitive ability (the ability to reflect on the nature or contents of one’s conscious awareness). In addition, younger adolescents may have more difficulty in self-regulating their behaviour (eg, when being confronted with challenging emotions/thoughts). As such, risk/resilience processes may differ between younger and older adolescents. Perhaps, this curriculum might be indicated in mid-adolescence when young people become more self-reflective and use metacognition.^24^ There is emerging evidence that older adolescents benefit from mindfulness curricula focused carefully on their needs and developmental stage.^25^ Moreover, there is evidence that in late adolescence mindfulness training is beneficial when people choose, rather than are required to, engage with mindfulness training.^26^

Consideration of the mental health status of young people also seems key. Adolescents with mental health needs did not benefit from this SBMT; indeed, it may be contraindicated for this group. More at-risk children are likely to have poorer executive function or develop these skills later. Consistent with other studies,^27^ low-intensity programmes may bring awareness to upsetting thoughts, feelings and mental health difficulties, but not provide sufficient support to enhance resilience, especially if such difficulties are social/societal. Findings of the MYRIAD trial showed no main effects on the primary outcomes,^8^ but our subgroup analyses suggest that more targeted and intensive interventions would be required for those with greater mental health needs.

With respect to implementation, the MYRIAD trial aimed for an adequate test of SBMT by ensuring that key implementation factors (eg, dose, quality, fidelity, reach and mindfulness practice) were optimal.^28^ We observed good fidelity and dose of our SBMT, considerable reach and an ‘advanced beginner’ quality of delivery. However, students’ engagement with the mindfulness practice during/after the intervention was strikingly low. There is growing acknowledgement that young people should codesign interventions to maximise accessibility, engagement and effectiveness. This refers not only to curriculum content but also to preferences for how to learn, and would likely be different at different developmental stages. Nevertheless, none of the mentioned implementation factors was significantly and directly related to students’ mental health and well-being at 1-year follow-up, potentially due to the low intensity of the SBMT—10 sessions in year 8 or 9, and four booster sessions the following year.

Consistent with one of the study’s main premises, improvements in mindfulness skills and executive function predicted our primary outcomes at 1-year follow-up. However, at least in its current format (.b programme), SBMT does not support students learning these foundational skills because the effects on these two skills were very small and had no clinical relevance. Reinforcing programme components with the aim of improving these skills could potentially increase the effectiveness of the intervention, although our findings may also be reflective of the natural developmental trajectory of these abilities in the sense that they may not be readily amenable to intervention. Perhaps other programmes may nonetheless find ways to support their development. Future studies should ask how best to support young people learning these skills.

The study had several limitations. It was a secondary analysis of a cluster RCT, and while the RCT was powered to observe intervention effects on the primary outcomes, a different sample size or replication would be needed to evaluate more complex interactions. In this sense, we have done a large number of statistical tests and have obtained no more statistically significant findings than we would expect if there were truly no associations. We used adolescent self-report which, while appropriate for some measures (eg, mental health assessed by well-established questionnaires), may have been less so for others (eg, mindfulness practice). Our measure of school reach only reflects the percentage of trial students receiving SBMT out of the year group, which is not reflective of the actual complexity, as there were also additional non-trial students who may have received SBMT. It is possible that observed effects may be bidirectional or indeed affected by other moderators and mediators. Nevertheless, we included a 1-year follow-up to temporally sequence possible chained effects. Finally, within the MYRIAD trial, the SBMT could be implemented as either additive or substitutive of an established SEL curriculum, and thus, the content and extent of SEL delivery could be different between schools. The study also has several strengths. So far, this is the largest cluster RCT evaluating an SBMT programme, and it was tested against social–emotional TAU in line with good practice; all participating schools had a strategy and structure in place for delivery of adequate SEL curricula. The external validity was maximised by a representative sample of students in secondary schools in the UK, and attention to implementation factors. Finally, this work was possible because we measured key dimensions in our theory of change (figure 1A).

## Clinical implications

It has been observed that a universal intervention can still be useful even if there is no overall effect, for example, via positive effects for some subgroups. However, in the MYRIAD trial, we have found potential iatrogenic effects for those participating students with existing or emerging mental health difficulties. This questions the use of this SBMT curriculum as a universal intervention. Given the substantial differences in school systems around the world, future research might explore whether different universal SEL curricula generally and SBMT curricula specifically might be appropriate in different settings. Moreover, future research and innovations should carefully consider the unique needs and developmental stage of young people.

## Data Availability

Data are available upon reasonable request. The data and codebook from the MYRIAD Trial are available from Prof Kuyken (willem.kuyken@psych.ox.ac.uk) upon request (release of data is subject to an approved proposal and a signed data access agreement).
