# Supplementary material for: School-based mindfulness training in early adolescence: what works, for whom and how in the MYRIAD trial?
Source: Evid Based Ment Health. 2022 Jul 7;25(3):117–24. doi: 10.1136/ebmental-2022-300439 (PMC9340034; doi:10.1136/ebmental-2022-300439)
Supplement: Supplementary data [file ebmental-2022-300439supp001.pdf]

School-based mindfulness training in early adolescence: what works, for whom, and how in the MYRIAD Trial?

Supplements

Supplement A: School-based mindfulness training (SBMT) and Teaching as Usual (TAU) (page 3)

Supplement B: Details of the measures (page 6)

Supplement C: Details of the latent profile analysis (LPA) procedure (page 18)

Supplementary Table S1: Baseline descriptive of potential moderators by trial arm status and overall using the sub-groups through which moderation results are presented for descriptive purposes (page 19)

Supplementary Table S2: Data availability (page 20)

Supplementary Table S3: Selected study/trial student baseline characteristics by post-intervention status (page 21)

Supplementary Table S4: Selected study/trial student baseline characteristics by 1 year follow-up status (page 22)

Supplementary Table S5: Subgroup analyses of risk for depression at 1-year follow-up (page 23)

Supplementary Table S6: Subgroup analyses of social-emotional-behavioural functioning at 1-year follow-up (page 24)

Supplementary Table S7: Subgroup analyses of well-being at 1-year follow-up (page 25)

Supplementary Table S8: Subgroup analyses of risk for depression at 1-year follow-up (page 26)

Supplementary Table S9: Subgroup analyses of social-emotional-behavioural functioning at 1-year follow-up (page 27)

Supplementary Table S10: Subgroup analyses of well-being at 1-year follow-up (page 28)

Montero-Marin J, *et al. Evid Based Ment Health* 2022; 25:117–124. doi: 10.1136/ebmental-2022-300439

Supplement E: Model selection, latent moderator interpretation, and student classification according to the LPA (page 29)

Supplementary Figure S1: Graphical representation of the distribution of predictor variables between latent profiles (page 36)

Supplementary Table S11: Latent Profiles and Associated Baseline Characteristics by trial arm (page 37)

Supplementary Figure S2: Risk for depression, social-emotional-behavioural functioning, and well-being at each time point by latent profile and group (page 38)

Supplementary Table S12: Subgroup analyses of primary outcomes at post-intervention and 1-year follow-up by latent profile (high/low risk) moderator (page 39)

Supplementary Table S13: Descriptive data for the implementation factors in the intervention arm (page 40)

Supplementary Table S14: Instrumental Variable Analysis of outcomes at 1-year follow-up, with allocated group as an instrument for quality after being dichotomised (page 41)

Supplementary Table S15: Instrumental Variable Analysis of outcomes at post-intervention, with allocated group as an instrument for the implementation variables (page 42)

Supplementary Table S16: Instrumental Variable Analysis of outcomes at post-intervention, with allocated group as an instrument for quality after being dichotomised (page 43)

Supplementary Table S17: Raw descriptive data for the mediating factors by trial arm status and overall (page 44)

References for the Supplements (page 45)

Supplement A: School-based mindfulness training (SBMT) and teaching as usual (TAU)

School-Based Mindfulness Training (SBMT) programme (.b)

The School-Based Mindfulness Training (SBMT) programme was designed to teach mindfulness skills that support young people's resilience. It was adapted from mindfulness-based cognitive therapy,[1] and developed over more than 5 years by three classroom teachers (Richard Burnett, Chris Cullen, and Chris O'Neill) who are also experienced mindfulness practitioners. Their aim was to make it acceptable to young people across the full spectrum of functioning from mental health problems to flourishing, enabling all young people to use mindfulness skills to manage emotions, academic study, sport, sleep, and relationships. This included ensuring that the programme can be taught in mainstream schools, how best to engage hard-to-reach students, and how to manage challenging classroom behaviour.

The SBMT comprised several elements, delivered through the school curriculum, over several years, supported by teacher training. The bulk of the SBMT was taught to students in a set of 10 structured lessons (within the trial, taught in English years 8 and/or 9). Typically, the SBMT programme was delivered in the spring terms (January through April), with support to continue using these mindfulness skills into the summer term. In the following school years, there are four booster lessons intended to continue and support students' further learning and ongoing mindfulness practice (e.g., lunchtime clubs or drop-in sessions). This follow-on training in subsequent school years aims to sustain, deepen, and enhance students' learning, e.g., to managing tests and examinations, and to embed mindfulness in the school ecology/climate.

The SBMT includes a combination of psychoeducation and practical skills involved in training the mind, learned in an experiential way, through short mindfulness practices which focus on the breath, body, and immediate experience. Additional classroom discussion aims to support the application of new skills in everyday life. Its design aligns with principles identified as important for its effectiveness in several reviews of school-based programmes that promote mental health and well-being and teach social and emotional competence. These principles include: explicitly teaching skills and attitudes; tailoring components and approaches to the needs of young people; using a range of age-appropriate, interactive, experiential and lively teaching methods; providing age-appropriate resources; e.g., in this context resources that bring mindfulness to life (including a course booklet, a set of online mindfulness exercises, and mindfulness practices that are introduced through animations and available as digital downloads); intensive, focused teacher education to build teachers' self-efficacy and well-being; and programme implementation which pays close attention to clarity and fidelity, in this case supported by a manual and indicative script.[2] Building on data that greater practice is associated with better outcomes,[3,4] the SBMT programme includes strategies to support teachers in keeping mindfulness integral to the culture of their year group/the school as a whole. Examples of good practice in this area could include teacher catch-up days/support events, suggested schedules for progressive, regular mindfulness input throughout year groups, suggested smartphone apps for both students and teachers, and using parts of the SBMT in core curriculum subjects.

Montero-Marin J, *et al. Evid Based Ment Health* 2022; 25:117–124. doi: 10.1136/ebmental-2022-300439

Our approach to implementing the SBMT was informed by theory and implementation science [5] and was designed to be fully integrated into the school curriculum, over several years. Because implementation may affect both reach and outcomes, all schools were supported with implementation guidance to increase the likelihood that it was introduced into the schools in ways that maintain its integrity and are sustainable. Implementation started with engaging the school's leadership team, and then identifying a potential pool of teachers from within the school who could be trained and timetabled to deliver it to the students. The selected teachers then went through a training programme (see below).

All participating schools randomised to SBMT agreed to deliver the SBMT programme to a minimum of three classes within years 8 and/or 9 or equivalent year groups across the nations (students aged 12-14, who were aged 11-13 at baseline). Schools were also encouraged to consider how they might introduce mindfulness into the curriculum more broadly, for the potential benefit of other school students and the wider school climate.

The SBMT teacher training involved first participating in an 8-week personal mindfulness-based cognitive therapy for life (MBCT-L) programme. MBCT-L was developed as a mindfulness training for the general population that supports resilience and well-being. The programme comprises eight 2-hour sessions per week, with an all-day mindfulness session supported by a course handbook and online mindfulness practices.[6] Participants are encouraged to develop a daily mindfulness practice, both during the training and to sustain this in an ongoing way afterwards. From the pool of teachers undergoing personal mindfulness training, schools selected a sub-sample of teachers to teach the SBMT to their students. Senior leadership teams in schools based their selection on whether teachers would be willing and available to attend further training and could be timetabled to teach the SMBT to participating study classes. Identified teachers attended a 4-day training workshop to learn how to deliver the SBMT curriculum to students. Following this 4-day training, teachers taught at least one full SBMT curriculum to non-study students, with support from an experienced mindfulness instructor, before teaching the SMBT to the study sample.

We have reported separately on the acceptability, effectiveness, and cost-effectiveness of this teacher training route,[7] as well as the relative merits of less and more intensive teacher training in terms of acceptability and effectiveness.[8] Based on the findings from this work the teachers received the more intensive mindfulness curriculum, as well as ongoing support to implement and deliver the SBMT curriculum to students.

Within participating schools, as many teachers as possible (up to 15) were encouraged to engage with the personal mindfulness training, to give schools the best opportunity to timetable the required number of teachers to teach the SBMT curriculum to study classes. Only teachers selected to teach study classes (usually 4 or 5 teachers) went on to complete the 4-day training workshop to learn about the delivery of the student SBMT curriculum. Training a larger group of teachers in SBMT was also intended to support embedding mindfulness within the school and provide a support network for those teachers who went on to teach the SBMT curriculum to students.

Teaching as usual (TAU)

The trial aimed to establish if SBMT, when integrated into social-emotional teaching in secondary schools, adds value over and above current good practice. Recent UK Department of Education reports suggest that 60% of secondary schools offer Personal, Social, Health and Economic Education (PSHE) lessons that are 'good or more', and that this provision is offered to students aged 11–16 years (Key Stages 3 and 4) through a variety of methods including regular scheduled lessons, drop-down days, within other subjects, and in tutor/form time.[9] Determining whether schools have good PSHE provision is challenging. In cohort 1, schools were eligible for inclusion if their provision of PSHE (or equivalent) met four criteria: (1) the presence of discrete, regular, named teaching time for PSHE, (2) a named PSHE lead, (3) a written PSHE policy, and (4) a named member of the senior leadership team responsible for PSHE.[10] However, for cohort 2, the 'written SEL policy' criterion was modified to "documentation denoting clear strategic planning of SEL within the school." Experience in cohort 1 indicated that schools do not always use the term 'SEL policy' to denote strategic planning of SEL. Moreover, in some cases, there are schools that have an extensive, well-established, and well-documented SEL curriculum, indicative of a clear structure and strategy around SEL, but do not have this formalised as a school policy.[11]

TAU schools agreed not to provide the MT programme (or other curricula that include MT) until study completion.

Supplement B: Details of the measures

Demographics and school/student characteristics

Student demographics, including gender (the original categories used were male, female, other, prefer not to say; however, because of the very small numbers of the two last categories, we only analysed male and female in the present study), ethnicity (White, Arab/Arab British, Black/African/Caribbean/Black British, Mixed/Multiple Ethnic Groups, Other Ethnic Group), were gathered via student self-report at baseline. Year group (year 7, year 8, year 9, year S1), and dates of birth were reported by the school, and the research group calculated the corresponding student's age in years.

School characteristics such as country (England, Scotland, Wales, and Northern Ireland), school size (<1,000 students vs. ≥1,000 students), type of school (mixed and girls only), Ofsted school quality rating (Does not require improvement: outstanding, good, and requires improvement: requires improvement, inadequate), school deprivation (% of students eligible for free school meals: in England, children living in households on income-related benefits (such as universal credit) are eligible for free school meals, as long as their annual household income does not exceed £7,400 after tax, not including welfare payments. This is the same in Wales and Scotland, however in Northern Ireland it is set at £14,000 a year –all the analyses that included this variable were carried out using its original distribution in a continuous way; however, for descriptive purposes only and to facilitate interpretation, we used the $M \pm 1SD$ as a cut-off criterion), urbanicity (urban vs. rural), were obtained at baseline. All school-level measures (e.g., type of school, school size, and urbanicity) were obtained using publicly available data published by the constituent nation the participating schools resided within. Data was usually obtained online from the education and statistics departments (e.g., Department of Education, England). Where information was not available, data was obtained through email correspondence with the department or school. In all cases, published publicly available data were collected referring to the year in which participating students provided baseline (T0) questionnaire data. The governmental defined urban/rural classification was used to define the urbanicity or rurality of the areas the participating schools resided within. School quality is measured differently in public and private schools and across the nations. Thus, we developed a measure that combines all the different school inspection rating systems into one measure, ranging from "outstanding" to "require improvement". Each constituent nation uses their own criteria and parameters most relevant to their nation to define urban versus rural areas. Dependant on the nation, this information is available in varying degrees of specificity. In order to draw direct comparisons between participating schools, the governmental defined dichotomous categorisation of schools was obtained and used for our analysis. We also described (a) the quality of 'Personal, Social, Health and Economic Education' (PSHE) provision, and (b) the school social-emotional learning (SEL) ethos.

Personal, Social, Health and Economic Education (PSHE) provision.

Social Emotional Learning (SEL) in England is taught as part of 'Personal, Social, Health, and Education' (PSHE) lessons (also called 'PSE', 'PSHCE', 'Health and Well-being', 'Health and Social Care', and 'Life lessons'). Due to the fact that delivering PSHE lessons in schools is not mandatory in England, there is considerable variation

Montero-Marin J, *et al. Evid Based Ment Health* 2022; 25:117–124. doi: 10.1136/ebmental-2022-300439

across schools in the delivery of PSHE lessons (in terms of content covered and teaching time allocated). A literature review[12] highlighted that there are no existing measures of PSHE to assess which schools had a minimum level of good practice in PSHE to be considered for study participation. Thus, a new PSHE assessment tool was devised for this study. For inclusion in the trial, schools had to meet 5 criteria for their current PSHE provision: regular, discrete, named teaching time for PSHE (or equivalent); a designated PSHE lead; a named member of the Senior Leadership Team (SLT) responsible for PSHE; documentation denoting clear strategic planning of SEL within the school; and evaluation of students' progress in PSHE. Once schools became a participating trial school, PSHE was assessed by discussing PSHE provision with the teacher responsible for PSHE at each school (or a member of the Senior Leadership Team). Sixteen quality indicators (listed below) were used to assess PSHE provision. They were created specifically for this trial and identified through a review via expert consultation.[9] Schools were assigned a score (out of 16) reflecting the number of quality indicators present (subscale scores indicate quality in the domains of Leadership and Strategic Approaches to PSHE, Curriculum Content and Delivery, and Assessment, Evaluation, and Consultation). Total scores were used in the present study. The items used, organised by sub-scales, were the following:

Sub-scales	Indicators (score)
Leadership and Strategic Approaches to PSHE from Consensus Indicators	A designated PSHE lead (0 = no, 1 = yes)<br>A named member of SLT has responsibility for supporting PSHE (0 = no, 1 = yes)<br>A written PSHE policy (0 = no, 1 = yes)<br>School's own rating of the quality of its PSHE provision (0-4 = 0, 5-10 = 1)<br>PSHE provision is part of the school improvement plan (0 = no, 1 = yes)<br>How well informed does the PSHE lead feel about local PSHE education CPD opportunities (0-4 = 0, 5-10 = 1)
Curriculum Content and Delivery from Consensus Indicators	Regular discrete, named teaching time for PSHE, including drop down days or tutorial time (0 = no, 1 = yes)<br>PSHE lead teaches PSHE lessons (0 = no, 1 = yes)<br>Topic Coverage KS3 and KS4 - School provides coverage of all elements of PSHE curriculum (0 = no, 1 = yes)<br>PSHE lead involved in planning: evidence of attempts to plan and coordinate PSHE across KS3 and KS4 (0 = no, 1 = yes)<br>Teaching Methods Used: School uses at least 6/10 methods for delivering PSHE (0 = no, 1 = yes)
Methods of Assessment, Evaluation and Consultation from Consensus Indicators	Any evaluation of student progress in PSHE (0 = no, 1 = yes)<br>Informal feedback (0 = no, 1 = yes)<br>Student / peer assessment of feedback (0 = no, 1 = yes)<br>Written feedback on student's progress reports (0 = no, 1 = yes)<br>School uses feedback to plan PSHE (0 = no, 1 = yes)

School social-emotional learning (SEL) ethos.

The concept of school 'ethos' and culture has been previously proposed to describe the underlying values and attitudes that the school represents, specifically in relation to the way staff and students relate, as well as the development of bonds between youth and adults, and the opportunities for participation in positive social activities.[13] In the field of preventing mental disorder, it has been said that school ecology should be a central focus of attention.[14] Following this, we proposed the 'school social-emotional

learning (SEL) ethos' construct, a new measure developed and evaluated in the MYRIAD study, as the school's commitment to and progress towards mental health and well-being. In order to derive a measure of the broad SEL ethos construct, we proposed gathering existing data from various relevant sources at baseline identifying all those variables that map onto the hypothesized latent construct of school SEL ethos in relation to promoting students' social, emotional, and mental well-being. The following school-level measures were considered: our PSHE assessment tool total score; a school ecology total score measure aggregated from averaged teacher ratings based on the teacher version of the "School Climate and Connectedness Survey"[15] that included the sub-scales of 'school leadership and involvement', 'staff attitudes' and 'respectful climate'; Ofsted school quality rating, and an independent researcher rating of the school's commitment to SEL based on the direct observation of the school. Firstly, all the measures were re-scaled to a new range from 0 to 4 points to ensure that all the variables contributed equally to the computation of the final index. After this, Pearson's r correlations were calculated (they ranged between 0.22 and 0.58). The characteristics of this correlation matrix were: KMO = 0.64; Barlett's test = 71.5 (df = 6; $p < 0.001$), determinant = 0.39. Optimal implementation of parallel analysis was used as a dimensionality test to decide on the number of factors to be retained. The number of random correlation matrices used was 500 and the generation of random correlation matrices was based on the permutation of sample values. The advised number of dimensions was 1 when the mean of random percentage of variance was considered, which explained a total of 65% of real-data variance. The robust unweighted least squares (RULS) method, correcting for robust mean and variance adjusted chi-squared statistic, was employed for factor extraction, using the correlation matrix as data entry. The one-dimensional structure produced loadings between 0.54 and 0.67. The factor determinacy index had a value of 0.85 and marginal reliability showed a value of 0.72 (factor scores were calculated by means of Bayes Expected a Posteriori –EAP– estimates transformed to T-scores). Construct replicability obtained a value of H = 0.72. The omega composite reliability for the unidimensional factor also obtained a value of 0.72. The valence of the factorial scores means that the construct measures "SEL school ethos", and therefore higher scores represent a more conducive school ethos towards the promotion of social, emotional, and mental well-being. All the analyses that included this variable were carried out using its original distribution in a continuous way; however, for descriptive purposes only and to facilitate interpretation (e.g., moderation analyses), we used the M±1SD as a cut-off criterion.

Co-primaries

The following three primary outcomes were assessed at baseline, pre-intervention, post-intervention, and 1-year follow-up.

Center for Epidemiologic Studies for Depression Scale (CES-D).

The "Center for Epidemiologic Studies for Depression Scale" (CES-D)[16] is a 20-item questionnaire that assesses depressive symptoms in the past week (e.g., "I felt depressed"), and it has been validated for the use with adolescents.[16] Each item is rated on a rating-scale from 0 ("rarely or none of the time") to 3 ("most or all of the time"), yielding a total score that ranges between 0 and 60, with higher scores meaning greater risk for depression. All the analyses that included the CES-D were carried out

Montero-Marin J, *et al. Evid Based Ment Health* 2022; 25:117–124. doi: 10.1136/ebmental-2022-300439

using its original distribution in a continuous way. Nevertheless, two cut-off points have been proposed for the CES-D: a) a lower cut-off point of 16 and above to identify students at risk of depression,[17] and b) a higher cut-off point of 28 and above to identify students with symptoms likely to meet diagnostic criteria for major depressive disorder.[16] For descriptive purposes only, and to facilitate interpretation, these two validated criteria were used in order to categorise participants into three sub-groups (‘low’: < 16; ‘at risk’: 16-27; and ‘caseness’: ≥ 28). The internal consistency value (Cronbach’s alpha) of the CES-D in our study was α = 0.88 at baseline, α = 0.91 at pre-intervention, α = 0.92 at post-intervention, and α = 0.92 at 1-year follow-up.

Strengths and Difficulties Questionnaire (SDQ).

The “Strengths and Difficulties Questionnaire” (SDQ)[18] is a 25-item questionnaire that assesses social, emotional, and behavioural strengths and difficulties over the previous 6 months (e.g., “I am constantly fidgeting or squirming”). Each item is rated on a rating-scale from 0 (“not true”) to 2 (“certainly true”). The SDQ’s five sub-scales assess emotional symptoms, conduct problems, hyperactivity/inattention, peer problems and pro-social behaviour. The total difficulties score measures social-emotional-behavioural functioning (range 0-40), and is derived by summing the first four subscales, where higher scores indicate greater difficulties. Higher scores on the pro-social subscale, in contrast, indicate better social functioning (although subscale scores have not been used in the present study). All the analyses that included the SDQ were carried out using its original distribution in a continuous way. Nevertheless, SDQ total scores can be interpreted using either the 4-band categorisation; ‘close to average’ (0-14), ‘slightly raised’ (15-17), ‘high’ (18-19) and ‘very high’ (20-24); or the 3-band categorisation, ‘normal’ (0-15), ‘borderline’ (16-19), and ‘abnormal’ (20-40).[19] For descriptive purposes only, and to facilitate interpretation, the 3-band categorisation was used in the present study. The SDQ was completed by students themselves, with an internal consistency (Cronbach’s alpha) of α = 0.83 at baseline, α = 0.84 at pre-intervention, α = 0.85 at post-intervention, and α = 0.85 at 1-year follow-up.

Warwick-Edinburgh Mental Well-being Scale (WEMWBS).

The “Warwick-Edinburgh Mental Well-being Scale” (WEMWBS)[20] is a 14-item measure assessing both feeling and functioning aspects of mental well-being over the last two weeks (e.g., “I’ve been feeling useful”). Items are scored on a rating-scale from 1 (“none of the time”) to 5 (“all of the time”), yielding a total score that ranges between 14 and 70. Items are worded positively and therefore higher scores indicate greater levels of mental well-being. The WEMWBS measure has been validated for its specific use in adolescents.[21] All the analyses that included the WEMWBS were carried out using its original distribution in a continuous way. There are no established cut-offs for the WEMWBS. However, for descriptive purposes only and to facilitate interpretation, we used the M±1SD as a cut-off criterion, as previously suggested (see https://warwick.ac.uk/fac/sci/med/research/platform/wemwbs/). The internal consistency of the WEMWBS in our study was α = 0.88 at baseline, α = 0.87 at pre-intervention, α = 0.89 at post-intervention, and α = 0.91 at 1-year follow-up.

Implementation factors

Fidelity to the original SBMT programme.

The fidelity to the original ".b" SBMT programme was measured as the percentage of the standardised curriculum that was covered overall in two randomly selected lessons per intervention class. All SBMT lessons were filmed and a subset of 2 out of the 10 possible lessons from each class were evaluated. The two lessons evaluated for each class were randomly chosen by a computer random number generator from a subset of combinations which were chosen as they provided the best opportunities of observing full practice (these possible combinations were: 3&6, 3&7, 4&6, where available; whenever these classes were not available, e.g., not recorded, other appropriate classes were reviewed). Teachers did not know in advance that these combinations would be chosen. For each randomly selected lesson, independent evaluators who were experienced mindfulness instructors indicated whether key curriculum elements (essential and non-essential, as they are defined by the ".b" SBMT teaching materials), were delivered or not. These ratings were summarised as the percentage of curriculum elements covered per lesson, and they were averaged across the two ".b" randomly selected lessons to provide a percentage of elements covered per intervention class. The following "*MiSP .b Fidelity Checklist*" was used by the two independent evaluators:

Below is a breakdown of elements in each of the 10 lessons that make up the .b curriculum. If the element is included in the lesson observed, please place a tick in the relevant column; leave blank if it is not. If there is lack of clarity around this, for example, an element is described but not fully or accurately, please comment in the final column.

Nb. For research purposes, essential elements have been distinguished from non-essential ones with an arrow. This is based on the .b teacher's notes, which depict an image of 'stickman' in the corner of the page, to inform teachers which elements of the curriculum MUST be included for the course to constitute the .b curriculum.

Lesson observed:

**Introduction**	**Element included**	**Comment**
Introduce 'possibilities' associated with mindfulness		
Introduce neuroplasticity and brain training (e.g., black cab)		
Experiential introduction to mindfulness (e.g., Mindfulness of hands practice)		
Mind/body connection		
Definition of mindfulness (kung fu panda)		
Brief introduction to .b practice		
Mindfulness and difficulties		
Mindfulness and concentration		
Mindfulness and flourishing (sport, music, business, spirituality)		
Student quotes		.
Strong silence		
Lesson overview		
Highlight importance of home practice		
Closing .b		
**Lesson 1**	**Element included**	**Comment**
Possibilities revisited		

Montero-Marin J, *et al. Evid Based Ment Health* 2022; 25:117–124. doi: 10.1136/ebmental-2022-300439

Ground rules		
Explanation of 'Searchlight of attention' (using torch)		
Play attention practice		
Attention - puppy comparison		
Animation		
Puppy Training practice		
Finger breathing practice and inquiry		
Home Practice		
**Lesson 2**	**Element included**	**Comment**
Home practice review		
'My mind feels, my body feels' practice		
Animal minds activity		
Animation		
Clear definition of Attitude (curiosity, kindness, patience, allowing etc)		
FOFBOC practice		
Inquiry		
Didactic link made between mind state, attitude and FOFBOC (dropping the anchor)		
Home practice		
**Lesson 3**	**Element included**	**Comment**
Home practice review		
Identify general worries for age and stage		
7/11 practice		
Two modes of mind		
Link between story telling mind and sleep disturbance		
Story telling mind activity – four sentences		
Scenario – demonstrate 'hot cross bun' from examples given		
Animation		
Overthink, rumination, snowball explanation.		
Beditation practice		
Inquiry		
Home practice		
**Lesson 4**	**Element included**	**Comment**
Animation		
Autopilot explanation		
Chocolate Practice		
Inquiry		
Introduction of Chilli (life's not always wonderful)		
Victor Frankel		
Explanation of reactivity (tiny tingles) and impact of mindfulness creating a gap between stimulus and response		
Chilli practice		
Inquiry		
.b practice		
Home practice		
**Lesson 5**	**Element included**	**Comment**
Home practice review		
Introduction to moving mindfully		
Animation		
Standing practice		
Inquiry		
Explanation of 'flow' or being 'in the zone'		
Nicola Benedetti clip		
.b Practice (importance of non-striving)		
Last Samurai clip		
Modes of mind		
Mindful walking practice		
Introduce routine activities		
Home practice		
**Lesson 6**	**Element included**	**Comment**
Home practice review		

Introduction to concept of 'thought traffic' and relationship to thought (standing back)		
Description of metacognition		
Animation		
Sounds and thoughts practice		
Inquiry		
Description of physical brain, neurons, and synaptic connections – link to roads/motorways (frequent thoughts = wider roads)		
Identify common thought buses activity		
Staying at the Bus Stop practice		
Inquiry		
Alternative analogies		
Home practice		
**Lesson 7**	**Element included**	**Comment**
Introduce topic – dealing with difficult emotions		
Introduce react v respond		
In what situations do you feel stress?		
Hot cross bun - what happens when you get stressed?		
Animation		
Description of brain activity related to stress		
Physical reactions to threat in the body		
Long term health implications of stress		
Describe fight / flight and difference in modern day stressors		
Stress induction practice (including .b)		
Inquiry		
Draw stress signature		
Campfire time		
Guesthouse poem		
Home practice		
**Lesson 8**	**Element included**	**Comment**
Introduction (mentioning the term 'heartfulness')		
Grape practice		
'Since the day you were born….' Activity		
Auschwitz discussion		
Alice Hertz-Sommer video		
Animation		
Gratitude practice		
Inquiry		
Soul pancake clip		
Home practice		
**Lesson 9**	**Element included**	**Comment**
Introduction		
Course review (either quiz or reflective)		
Sunscreen Video		
Reflective practice (what do I want to take with me)		
Write a letter (advice to future self)		
Questionnaire		
Certificates		
Next steps		

Dose - Number of SBMT sessions that students received.

The number of ".b" sessions that students attended (which ranged between 0 and 10 sessions) was completed by the ".b" teacher teaching the lesson, who reported the students' attendance of the SBMT lessons.

Quality - MBI-TAC (Teach).

To assess the quality of the SBMT intervention we considered the teaching competency. All SBMT lessons were filmed and a randomly selected subset of 2 out of the 10 possible lessons from each class were rated using the "Mindfulness-Based Interventions – Teaching Assessment Criteria" (MBI-TAC).[22-24] External evaluators did not see the students but only the teachers (students were not in frame but were anonymised if appearing by accident by blurring them out to just show the teachers; this was so because pupils were not meant to be on film, so they were anonymised in the case of filming them accidentally). Lessons were rated by one of four different assessors using an adapted version of the MBI-TAC for the teaching context (MBI-TAC-Teach, see https://mbitac.bangor.ac.uk/documents/MBI-TAC-for-schools.pdf). External evaluators were MBI teachers, who had a recognised mindfulness training pathway, were qualified to teach the SBMT (".b") with more than two years of experience (and thus were familiar with the corresponding curriculum and issues in teaching the curriculum) and were qualified classroom teachers. They were trained in the use of the MBI-TAC-Teach assessment, by taking part in two days of training where the MBI-TAC-Teach was introduced by two experienced supervisors. They all had experience of being rated by the MBI-TAC in their own teaching pathway so all of them were familiar with this tool. The training focused on the aspects of the MBI-TAC-Teach tool, and the training was based on collective discussions and evaluations of some case studies to ensure standardisation. Evaluators were also allowed time to rate some examples independently to ensure consistency and that these ratings were within an acceptable range for all the evaluators at the end of the training. All evaluators took part in regular supervision sessions with the aim of ensuring/maximising assessment standardisation. A randomly chosen 'back-up' lesson was also used by the evaluators if they felt that observing the first two lessons did not provide sufficient evidence for the overall ratings. Certain lessons were not used, for example, lesson 5 ('Moving Mindfully') as students move around in this lesson meaning that it was not easy to capture the student and teacher interactions on film. If videos were not available for the chosen combination of lessons, then a decision was made to use different lessons based upon the videos available and the lessons that would provide the best opportunity to observe all domains. The MBI-TAC was developed in the context of Mindfulness-Based Stress Reduction and Mindfulness-Based Cognitive Therapy and was adapted to MBI-TAC (Teach) to be used to rate classroom teachers, teaching mindfulness to young people in school contexts. Competence is rated across 6 domains on a 6- point scale (1 = "incompetent", 2 = "beginner", 3 = "advanced beginner", 4 = "competent", 5 = "proficient", and 6 = "advanced"). The domains assess: coverage, pacing and organisation of session curriculum; relational skills; embodiment of mindfulness; guiding mindfulness practices; conveying course themes through interactive enquiry and didactic teaching; and holding the group learning environment. Evaluators provided competency ratings on the 6 domains, and an overall competency rating per lesson (based on their own overall assessment rather than a sum score of the 6 domains), for the two randomly selected lessons per intervention class. Based on the two lessons, an overall rating per domain for that class was completed, and then used by evaluators to provide one overall final competency rating per class as a measure of the quality of the intervention delivery. The following checklist for the .b curriculum was used for this study:

**Domain 1: Coverage, pacing and organisation of session curriculum**

-The curriculum is presented in a clear and engaging way in accordance with the instructions in the teacher manual<br><br>-The teacher adapts well to any constraints under which s/he is working (e.g., time of lesson, size and lay out of classroom, black out blinds etc.)<br><br>-Teacher is able to balance the goals of the lesson while responding to spontaneous events
**Domain 2: Relational skills**<br><br>-Teacher is mindful of the boundaries about how appropriate it is for students to share more personal thoughts and feelings in a classroom context, given the lack of confidentiality in a school environment. For example, knows when to stop them for their own well-being.<br><br>Students with special educational needs and disability (SEND) or behavioural issues are skilfully managed in the classroom. For example, a student with attentional and/or behavioural difficulties is able to learn as much as possible from the course whilst causing minimal disruption to the class dynamic. If they are unable to participate, the teacher skilfully deals with the situation by, for example, from encouraging them to quietly get on with another activity, or rest their head on desk and sleep, or quietly listen to music on headphones. This is with a view to enabling the learning of the whole class.
**Domain 3: Embodiment of mindfulness**<br><br>-Teacher knows when to mindfully step in and out of 'teacher mode' if disruptive behaviour is impeding the progress of the lesson and class control is becoming an issue. Teacher shows willingness to be firm and use sanctions, without losing their presence and sense of perspective.<br><br>The teacher is able to communicate their confidence and trust in mindfulness, even in the face of doubt, scepticism or resistance in the group.
**Domain 4: Guiding mindfulness practices**<br><br>-The appropriate schools-based MT in schools practices are taught in ways that children can understand and use them.<br><br>-Practices are guided in the way teachers were trained to guide practices, using appropriate vocabulary for this age group.<br><br>-Practices are guided from the experience of the teacher participating in practice with the group but maintaining appropriate visual contact as the group requires.<br><br>-The teacher moves around the classroom skilfully when required, e.g., to gently wake up a snoring student, or to move closer towards a giggling or misbehaving clutch of students.<br><br>-Teachers evidence good judgment in knowing how to manage students' sleepiness/wakefulness. This includes knowing the students and how to manage posture in terms of wakefulness/sleepiness. It may sometimes be appropriate for students to be encouraged to have their heads stay up as they practice and equally it may sometimes be appropriate when exhaustion prevails to allow them to rest their heads on their desks and be allowed to sleep.
**Domain 5: Conveying course themes through interactive inquiry and didactic teaching**

-Teacher demonstrates understanding of the curriculum and is able to relay this through the curriculum<br><br>-Teacher knows how to contextualise and make relevant any PowerPoint images, words, or concepts which the class may not immediately understand or connect with.<br><br>-Teacher does not introduce extraneous activity but may show initiative in bringing the curriculum to life in line with its aims and intentions through examples or activities.<br><br>-The teacher uses any aides (e.g., PowerPoint, video and props) skilfully such that they support the teaching rather than lead it. Whenever possible, general learning is 'drawn out' of students rather than 'telling' them. However, there are passages in the lessons that are more didactic, particularly when introducing a new idea.
**Domain 6: Holding the group learning environment**<br><br>-The teacher is aware of necessary/relevant information about individuals' needs in this group to be sure that they are able to respond to the group in skilful ways.<br><br>-The teacher is aware of the setting's safeguarding procedures and uses those appropriately, as necessary. For example, the teacher might find ways of directing a student to other sources of help within the school if appropriate.<br><br>-Classroom management appropriately supports delivery of the mindfulness training. Strategies to engage and support individuals to participate as fully as possible are used where these complement the approach of the mindfulness lessons. For example, the teacher is able to adapt his/her teaching style in whichever way is required to 'hold' the group. Sometimes this may be gentle, patient and receptive (the 'guide on the side' mode); at other times it may be entertaining, even eccentric, and faster-paced (the 'sage on the stage' mode); at other times the teacher may have to shift into a stricter and more authoritative mode to 'hold' a misbehaving class, so that mindfulness training is feasible, and then know how to shift back into a more classical mindfulness teacher mode without losing balance.<br><br>-The teacher transitions well between different phases of a lesson (e.g., from a more didactic phase, into a video clip, into a practice, out of the practice into pair work etc.). This includes the ability to orchestrate difficult whole class transitions quickly and smoothly (e.g., in Lesson 3 getting all the students out of their chairs and onto the floor or getting them up and standing in a circle in Lesson 7 if using 'shock ball').<br><br>-Teacher uses discipline skilfully when required. S/he does so in a way which is in line with the school's disciplinary procedures and child safeguarding policies. Once discipline is used, the teacher is able to move on with the lesson without being too 'thrown' by the previous, necessary intervention.<br><br>-Teacher maintains sense of humour and perspective throughout. If the students are not in the mood and everything s/he has tried to get them into the mood for practice has failed, s/he accepts this, tears up the lesson plan and salvages what s/he can.

Student reach.

Program reach (participation rates, program scope) refers to the rate of involvement and representativeness of program participants, and it is concerned with questions relating to the percentage of the eligible population who took part in the intervention, and their characteristics. In the present study, student reach was considered at post-intervention as

the proportion (%) of students attending more than 67%[25] of ".b" SBMT lessons relative to the study's year group (grade level) school student population.

Mindfulness practice.

We assessed the extent (i.e., frequency) of student home-based mindfulness practice during the SBMT programme, and after the SBMT programme, using a 6-item rating-scale, including the following items:

Item nº 1	"During the course you were taught a range of mindfulness practices. How often did you practice being mindful?"
Item nº 2	"During the course you were invited to pause and focus on your breathing by doing a 7-11 or FOFBOC or a .b (i.e., stop, breathe and be). How often did you do this?"
Item nº 3	"During the course you were taught to use 'beditation' as a way of helping you get to sleep. How often did you do this?"
Item nº 4	"During the course you were asked to be mindful in your everyday lives, for example walk a short distance mindfully, or eat a mouthful of food mindfully. How often did you do this?"
Item nº 5	"During the course you were asked to notice stress in your body, e.g., 'stress signature' in difficult times, noticing where in the body you were feeling stress. How often did you do this?"
Item nº 6	"During the course you were taught to think about your thoughts as passing objects such as buses, clouds or rivers that pass through your mind. How often did you do this?"

Items were answered at post-intervention (to respond to the frequency of mindfulness practice during the SBMT programme), and at 1-year follow-up (to respond to the frequency of mindfulness practice after the SBMT programme), on a Likert-type scale, ranging from 0 = "never", to 5 = "almost every day". Total scores were calculated by summing all the items divided by the number of items (i.e., mean total scores, that ranged from 0 to 5). Therefore, higher scores represent a higher frequency of student home-based mindfulness practice. The internal consistency (Cronbach's alpha value) of this measure of student engagement with the mindfulness practice was $\alpha = 0.89$ at post-intervention and $\alpha = 0.89$ at the 1-year follow-up measurement.

Mediators

Child and Adolescent Mindfulness Measure (CAMM)

The "Child and Adolescent Mindfulness Measure" (CAMM)[26] is a self-report measure of mindfulness skills designed for use with children and adolescents. It consists of 10-items, which measure awareness of the present moment as well as non-judgemental and non-avoidant responses to thoughts and feelings (e.g., "I keep myself busy, so I don't notice my thoughts or feelings"). Participants are asked how often each sentence is true, and responses are given using a 5-point Likert-type rating-scale, ranging from 0 ("Never True") to 4 ("Always True"). Each item is reverse scored and summed, producing a total score of 0-40, with higher scores corresponding to higher levels of mindfulness. The CAMM has been validated for use in non-clinical samples of adolescents,[27,28] and has adequate psychometric properties.[26] The internal

consistency (Cronbach's alpha value) of this measure of mindfulness skills was α = 0.84 at pre-intervention, α = 0.86 at post-intervention, and α = 0.88 at 1-year follow-up.

Behaviour Rating Inventory of Executive Function, Second Edition (BRIEF-2)

The "Behaviour Rating Inventory of Executive Function, Second Edition" (BRIEF-2)[29] is a 55-item self-report measure designed to assess self-perception of everyday behaviours associated with executive function in older children and adolescents (aged 11-18), e.g., "I have trouble sitting still". The BRIEF-2 assesses executive function across the past 6 months and takes into account the following seven domains: inhibit; self-monitor; shift; emotional control; task completion; working memory; and plan/organize. Items are rated as follows: 1 = "never", 2 = "sometimes", and 3 = "often". Total scores are calculated by summing the corresponding sub-scores, with higher scores suggesting higher levels of executive dysfunction. The three items of the infrequency scale ("I forget my name", "I have trouble counting to three", and "I cannot find the front door of my home") are only used as indicators of validity and are not included in the calculation of raw scale scores, so that the total score ranges between 52 and 156; higher scores indicate worse executive functioning. The student's self-report of the BRIEF-2 inventory was used in the present study. The internal consistency (Cronbach's alpha value) of the BRIEF-2 total score was α = 0.97 at pre-intervention, α = 0.97 at post-intervention, and α = 0.97 at 1-year follow-up.

Supplement C: Details of the latent profile analysis (LPA) procedure

First, the moderator was defined by LPA reflecting subgroups of children with particular patterns across the nine baseline characteristics that were included in the traditional subgroup (i.e., moderation) analysis, covering student characteristics (age, gender, ethnicity, social-emotional-behavioural functioning, risk for depression, and well-being), the school's broader context (school urbanity), school community (school deprivation), and school operational features (school social-emotional learning (SEL) ethos).[30] LPAs were conducted using maximum likelihood estimation with cluster (students within schools) robust standard errors. In principle, we were interested in classes that are optimally separated and are more likely to reflect 'true' classes in the population, more than in the full spectrum of heterogeneity. For that, we evaluated a series of LPA models containing one to eight latent profiles in a randomly selected sub-sample (split-half). To validate the structure of the selected latent profile model, we tested LPA models in the second half of the sample, and all subsequent analyses were then developed with the total sample. For model selection, we used the Akaike information criterion (AIC), consistent Akaike information criterion (CAIC), bayesian information criterion (BIC), sample-size-adjusted BIC (sBIC), Lo–Mendell–Rubin adjusted likelihood ratio test (LMR-LRT), as well as bootstrapped likelihood ratio test (BLRT), and we also calculated the index of classification accuracy (Entropy).[31] Second, we assigned students into their most likely baseline profile based on BCH weights, using variables that reflected the measurement error of the latent profile variable.[32] Lastly, we estimated the differential impact of SBMT across the latent subgroups for the three primary outcomes of risk for depression, social-emotional-behavioural functioning, and well-being at post-intervention, and 1-year follow-up. For that, profile assignments were used to calculate a joint model that combined the measurement latent profile mixture model and the auxiliary model where the latent profile was a moderator of a mixed linear regression, clustering for schools, and adjusting for the covariates mentioned in the traditional moderation analysis (see main manuscript, and Figure 1c).

Montero-Marin J, *et al. Evid Based Ment Health* 2022; 25:117–124. doi: 10.1136/ebmental-2022-300439

Supplementary Table S1: Baseline descriptive of potential moderators by trial arm status and overall using the sub-groups through which moderation results are presented for descriptive purposes

Potential moderators	Subgroup	TAU	SBMT	Total
Student characteristics		N = 4,144	N = 4,232	N = 8,376
Age	*11 years*	1,739 (42.0)	1,771 (41.8)	3,510 (41.9)
	12 years	2,035 (49.1)	2,097 (49.6)	4,132 (49.3)
	13 years	370 (8.9)	364 (8.6)	734 (8.8)
Gender*	*male*	1,904 (46.9)	1,807 (43.5)	3,711 (45.1)
	female	2,159 (52.1)	2,350 (56.5)	4,509 (53.8)
Ethnicity	*white*	2,965 (73.2)	3,237 (76.5)	6,202 (74.0)
	other	1,083 (26.8)	908 (23.5)	1,991 (26.0)
Risk for depression	*low*	2,820 (68.1)	2,809 (66.4)	5,629 (67.3)
	at risk	925 (22.3)	987 (23.3)	1,912 (22.8)
	case	395 (9.5)	434 (10.3)	829 (9.9)
Social-emotional-behavioural functioning	*normal*	2,904 (71.4)	2,982 (71.6)	5,886 (71.5)
	borderline	611 (15.0)	588 (14.1)	1,199 (14.6)
	abnormal	554 (13.6)	595 (14.3)	1,149 (14.0)
Well-being	*low*	604 (14.7)	624 (14.8)	1,228 (14.7)
	medium	2,861 (69.5)	2,902 (68.9)	5,763 (68.8)
	high	654 (15.9)	688 (16.3)	1,342 (16.1)
School broad context		N = 41	N = 43	N = 84
Urbanity	*Urban*	35 (85.4)	36 (85.4)	71 (84.5)
	Rural	6 (14.6)	7 (16.3)	13 (15.5)
School community				
Deprivation status	*<3% FSM*	7 (17.1)	4 (9.3)	11 (13.1)
	3-22% FSM	28 (68.3)	33 (76.7)	61 (72.6)
	>22% FSM	6 (14.6)	6 (14.0)	12 (14.3)
School operational features				
School quality rating	*Requires improvement*	6 (7.1)	5 (6.0)	11 (13.1)
	Does not require improvement	37 (44.0)	36 (42.9)	73 (86.9)
SEL ethos	*low*	8 (19.5)	4 (9.3)	12 (14.3)
	medium	25 (61.0)	31 (72.1)	56 (66.7)
	high	8 (19.5)	8 (18.6)	16 (19.0)

Age was reported by 8,376 students. Gender (male, female) was reported by 8,220 students. Ethnicity (white, other) was reported by 8,193 students. Risk for depression was reported by 8,370 students. Social-emotional-behavioural functioning was reported by 8,234 students. Well-being was reported by 8,333 students. Figures are numbers (frequencies) and percentages. *The non-binary gender students were not included in the analysis of gender as a potential moderator due to the very small numbers.

Supplementary Table S2: Data availability

**Primary outcomes**	**pre–post-intervention**	**pre–1-year follow-up**
Risk for depression (CES-D)	7,561 (90.3%)	7,238 (86.4%)
Social-emotional-behavioural functioning (SDQ)	7,542 (90.9%)	7,225 (86.3%)
Well-being (WEMWBS)	7,572 (90.4%)	7,244 (86.5%)

	**T0** K = 84; N = 8376 (100%)		**T1** K = 84; N = 8072 (96.4%)		**T2** K = 84; N = 7588 (90.6%)		**T3** K = 84; N = 7263 (86.7%)	
	**Mean (SD)**	**Missing (%)**	**Mean (SD)**	**Missing (%)**	**Mean (SD)**	**Missing (%)**	**Mean (SD)**	**Missing (%)**
CES-D	13.5 (9.9)	6 (0.1)	15.6 (11.1)	18 (0.2)	16.6 (11.7)	27 (0.4)	16.9 (11.9)	25 (0.3)
SDQ	11.8 (6.5)	124 (1.5)	12.4 (6.6)	30 (0.4)	13.3 (6.9)	46 (0.6)	13.1 (6.8)	38 (0.5)
WEMWBS	49.7 (9.7)	43 (0.5)	49.1 (9.1)	14 (0.2)	47.9 (9.5)	16 (0.2)	47.6 (9.8)	19 (0.3)

CES-D is the "Center for Epidemiologic Studies for Depression Scale". SDQ is the "Strengths and Difficulties Questionnaire" (specifically, the Total Difficulties - self report scale was used). WEMWBS is the "Warwick-Edinburgh Mental Well-being Scale".

Supplementary Table S3: Selected study/trial student baseline characteristics by post-intervention status

Variables	Students lost to post-intervention*			Remaining students**		
	**TAU (N = 346)**	**SBMT (N = 453)**	**Total (N = 799)**	**TAU (N = 3,798)**	**SBMT (N = 3,779)**	**Total (N = 7,577)**
Gender†						
Female, n (%)	183 (54.1)	205 (46.7)	388 (49.9)	1,976 (53.0)	2,145 (57.7)	4,121 (55.4)
Male, n (%)	149 (44.1)	223 (50.8)	372 (47.9)	1,674 (44.9)	1,501 (40.4)	3,175 (42.7)
Other, n (%)	1 (0.3)	4 (0.9)	5 (0.6)	11 (0.3)	10 (0.3)	21 (0.3)
Prefer not to say, n (%)	5 (1.5)	7 (1.6)	12 (1.5)	64 (1.7)	62 (1.7)	126 (1.7)
Ethnicity – White††, n (%)	243 (71.9)	336 (76.9)	579 (74.7)	2,722 (73.4)	2,901 (78.2)	5,623 (75.8)
Age, mean (SD)	12.2 (0.6)	12.2 (0.6)	12.2 (0.6)	12.2 (0.6)	12.2 (0.6)	12.2 (0.6)
Starting Year group						
Year 7, n (%)	163 (47.1)	231 (51.0)	394 (49.3)	1,979 (52.1)	1,851 (49.0)	3,830 (50.5)
Year 8, n (%)	166 (48.0)	201 (44.4)	367 (45.9)	1,661 (43.7)	1,677 (44.4)	3,338 (44.1)
Year 9, n (%)	11 (3.2)	1 (0.2)	12 (1.5)	53 (1.4)	78 (2.1)	131 (1.7)
Year S1, n (%)	6 (1.7)	20 (4.4)	26 (3.3)	105 (2.8)	173 (4.6)	278 (3.7)
Depression (CES-D)†††, M (SD)	15.5 (10.5)	16.3 (10.9)	15.9 (10.7)	13.1 (9.7)	13.3 (9.8)	13.2 (9.8)
Social-emotional-behavioural functioning (SDQ) Total Difficulties – self report††††, M (SD)	13.3 (6.5)	13.5 (6.8)	13.4 (6.7)	11.6 (6.4)	11.6 (6.5)	11.6 (6.4)
Well-being (WEMWBS)†††††, M (SD)	47.7 (10.3)	47.8 (10.1)	47.7 (10.2)	49.8 (9.7)	50.0 (9.7)	49.9 (9.7)

* Defined as those students with missing data on all three primary outcomes at post-intervention.
** Defined as those students with at least one of the primary outcomes at post-intervention.
†Sample size in lost to post-intervention group: 777: intervention arm: 439; control arm: 338. Sample size in remaining student's group: 7,443: intervention arm: 3,718; control arm: 3,725.
†† Sample size in lost to post-intervention group: 775: intervention arm: 437; control arm: 338. Sample size in remaining student's group: 7,418: intervention arm: 3,708; control arm: 3,710
††† Sample size in remaining student's group: 7,571: intervention arm: 3,777; control arm: 3,794
†††† Sample size in lost to follow-up group: 784: intervention arm: 443; control arm: 341. Sample size in remaining student's group: 7,468: intervention arm: 3,728; control arm: 3,740.
††††† Sample size in lost to follow-up group: 797: intervention arm: 452; control arm: 345. Sample size in remaining student's group: 7,536: intervention arm: 3,762; control arm: 3,774.
CES-D: Center for Epidemiologic Studies for Depression Scale. SDQ: Strengths and Difficulties Questionnaire. WEMWBS: Warwick-Edinburgh Mental Well-Being Scale.

Montero-Marin J, *et al. Evid Based Ment Health* 2022; 25:117–124. doi: 10.1136/ebmental-2022-300439

Supplementary Table S4: Selected study/trial student baseline characteristics by 1 year follow-up status

Variables	Students lost to follow-up*			Remaining students**		
	**TAU (N = 572)**	**SBMT (N = 554)**	**Total (N = 1,126)**	**TAU (N = 3,572)**	**SBMT (N = 3,678)**	**Total (N = 7,250)**
Gender[†]						
Female, n (%)	283 (50.2)	279 (52.1)	562 (51.1)	1,876 (53.6)	2,071 (57.2)	3,947 (55.4)
Male, n (%)	266 (47.2)	243 (45.3)	509 (46.3)	1,557 (44.5)	1,481 (40.9)	3,038 (42.7)
Other, n (%)	2 (0.4)	5 (0.9)	7 (0.6)	10 (0.3)	9 (0.3)	19 (0.3)
Prefer not to say, n (%)	13 (2.3)	9 (1.7)	22 (2.0)	56 (1.6)	60 (1.7)	116 (1.6)
Ethnicity – White[††], n (%)	420 (74.7)	404 (75.8)	824 (75.3)	2,545 (73.0)	2,833 (78.4)	5,378 (75.8)
Age, mean (SD)	12.2 (0.6)	12.2 (0.6)	12.2 (0.6)	12.2 (0.6)	12.2 (0.6)	12.2 (0.6)
Starting Year group						
Year 7, n (%)	283 (49.5)	292 (52.7)	575 (51.1)	1,859 (52.0)	1,790 (48.7)	3,649 (50.3)
Year 8, n (%)	280 (49.0)	245 (44.2)	525 (46.6)	1,547 (43.3)	1,633 (44.4)	3,180 (43.9)
Year 9, n (%)	7 (1.2)	0 (0.0)	7 (0.6)	57 (1.6)	79 (2.2)	136 (1.9)
Year S1, n (%)	2 (0.4)	17 (3.1)	19 (1.7)	109 (3.1)	176 (4.8)	285 (3.9)
Depression (CES-D)[†††], M (SD)	14.7 (10.6)	16.3 (10.9)	15.5 (10.7)	13.1 (9.7)	13.2 (9.7)	13.1 (9.7)
Social-emotional-behavioural functioning (SDQ) Total Difficulties – self report[††††], M (SD)	13.2 (6.5)	13.8 (6.7)	13.5 (6.6)	11.5 (6.3)	11.5 (6.5)	11.5 (6.4)
Well-being (WEMWBS)[†††††], M (SD)	48.7 (10.4)	47.8 (10.1)	48.2 (10.2)	49.8 (9.6)	50.0 (9.6)	49.9 (9.6)

* Defined as those students with missing data on all three primary outcomes at 1 year follow-up.
** Defined as those students with at least one of the primary outcomes at 1 year follow-up.
[†] Sample size in lost to follow-up group: 1,100: intervention arm: 536; control arm: 564. Sample size in remaining student's group: 7,120: intervention arm: 3,621; control arm: 3,499
[††] Sample size in lost to follow-up group: 1,095: intervention arm: 533; control arm: 562. Sample size in remaining student's group: 7,098: intervention arm: 3,612; control arm: 3,486
[†††] Sample size in remaining student's group: 7,244: intervention arm: 3,676; control arm: 3,568
[††††] Sample size in lost to follow-up group: 1,104: intervention arm: 539; control arm: 565. Sample size in remaining student's group: 7,148: intervention arm: 3,632; control arm: 3,516.
[†††††] Sample size in lost to follow-up group: 1,120: intervention arm: 549; control arm: 571. Sample size in remaining student's group: 7,213: intervention arm: 3,665; control arm: 3,548.
CES-D: Center for Epidemiologic Studies for Depression Scale. SDQ: Strengths and Difficulties Questionnaire. WEMWBS: Warwick-Edinburgh Mental Well-Being Scale.

Montero-Marin J, *et al. Evid Based Ment Health* 2022; 25:117–124. doi: 10.1136/ebmental-2022-300439

Supplementary Table S5: Subgroup analyses of risk for depression at 1-year follow-up

Potential modifiers	Subgroup	TAU mean (SD)	N	SBMT mean (SD)	N	g (95% CI)	Adjusted mean difference (95% CI)	p-value for interaction
**Student characteristics**								
Age ‡	*11 years*	16.18 (13.20)	1,508	17.02 (13.72)	1,537	0.06 (-0.01 to 0.13)	0.82 (-0.13 to 1.77)	0.072
	12 years	16.92 (15.49)	1,754	16.87 (14.50)	1,820	0.00 (-0.07 to 0.06)	-0.08 (-0.93 to 0.78)	
	13 years	18.48 (13.94)	304	17.56 (12.42)	315	-0.07 (-0.23 to 0.09)	-0.37 (-2.43 to 1.68)	
Gender	*male*	13.52 (10.86)	1,620	13.58 (14.93)	1,545	0.01 (-0.07 to 0.07)	0.35 (-0.36 to 1.06)	0.680
	female	19.50 (16.44)	1,873	19.63 (21.38)	2,070	0.01 (-0.06 to 0.07)	0.15 (-0.93 to 1.22)	
Ethnicity	*white*	16.94 (14.62)	2,542	17.17 (17.02)	2,829	0.02 (-0.04 to 0.07)	0.32 (-0.44 to 1.07)	0.397
	other	16.29 (16.24)	939	16.38 (13.10)	777	0.01 (-0.09 to 0.10)	0.12 (-1.08 to 1.32)	
Risk for depression baseline ‡	*low*	14.06 (13.88)	2,459	13.91 (11.99)	2,497	-0.01 (-0.07 to 0.04)	-0.13 (-0.84 to 0.59)	0.423
	at risk	20.18 (11.96)	774	21.84 (12.11)	832	0.14 (0.04 to 0.24)	1.68 (0.48 to 2.88)	
	case	28.53 (13.78)	329	27.93 (12.74)	341	-0.05 (-0.20 to 0.11)	-0.20 (-2.20 to 1.79)	
**School broad context**								
Urbanity	*Urban*	16.83 (15.95)	3,025	17.18 (17.18)	3,072	0.02 (-0.03 to 0.07)	0.27 (-0.47 to 1.01)	0.653
	Rural	16.10 (16.74)	541	16.06 (17.63)	600	0.00 (-0.12 to 0.11)	1.60 (-1.14 to 4.34)	
**School community**								
Deprivation status ‡	*<3% FSM*	15.20 (15.03)	721	17.56 (23.31)	404	0.13 (0.01 to 0.25)	1.77 (-0.76 to 4.30)	0.148
	3-22% FSM	17.03 (15.75)	2,423	17.07 (17.48)	2,806	0.00 (-0.05 to 0.06)	0.04 (-0.76 to 0.85)	
	>22% FSM	17.39 (11.91)	422	16.12 (9.02)	462	-0.12 (-0.25 to 0.01)	-0.33 (-1.81 to 1.16)	
**School operational features**								
SEL ethos ‡	*low*	17.12 (11.19)	592	17.45 (16.37)	285	0.02 (-0.12 to 0.17)	0.43 (-1.91 to 2.77)	0.312
	medium	16.95 (16.58)	2,122	16.78 (16.46)	2,646	-0.01 (-0.07 to 0.05)	-0.19 (-1.02 to 0.64)	
	high	15.81 (16.34)	852	17.58 (20.14)	741	0.10 (0.00 to 0.20)	1.96 (0.36 to 3.56)	

Mixed-effects linear regressions with cluster-robust maximum likelihood estimation, including schools (clusters) as random effects and adjusted for the cohort, country, school size, school sex, and the outcome at baseline. 95% CI: 95% confidence interval. p: p-value associated with the interaction term. SEL: socio-emotional learning. FMS: free school meals. Number of clusters (schools) = 84. The non-binary gender students were not included in the analysis of gender as a potential moderator due to the very small numbers. ‡ These variables were included in the models in a continuous way (they are categorised for descriptive purposes only to aid interpretation), and p-values are presented accordingly. g: Hedges' g effect size calculated as the difference in raw means between trial arms divided by the pooled standard deviation. TAU: teaching as usual. SBMT: school-based mindfulness training.

Supplementary Table S6: Subgroup analyses of social-emotional-behavioural functioning at 1-year follow-up

Potential modifiers	Subgroup	TAU mean (SD)	N	SBMT mean (SD)	N	g (95% CI)	Adjusted mean difference (95% CI)	p-value for interaction
**Student characteristics**								
Age ‡	*11 years*	12.78 (7.76)	1,508	13.28 (9.80)	1,537	0.06 (-0.02 to 0.13)	0.31 (-0.23 to 0.85)	0.502
	12 years	13.04 (7.95)	1,754	13.14 (6.82)	1,820	0.01 (-0.05 to 0.08)	0.15 (-0.26 to 0.56)	
	13 years	13.60 (6.62)	304	13.02 (6.74)	315	-0.09 (-0.24 to 0.07)	0.12 (-0.79 to 1.03)	
Gender	*male*	11.86 (6.43)	1,616	11.96 (9.80)	1,539	0.01 (-0.06 to 0.08)	0.26 (-0.17 to 0.70)	0.815
	female	13.93 (9.51)	1,872	14.14 (10.91)	2,069	0.02 (-0.04 to 0.08)	0.13 (-0.38 to 0.64)	
Ethnicity	*white*	13.34 (8.56)	2,537	13.34 (10.09)	2,824	0.00 (-0.05 to 0.05)	0.14 (-0.27 to 0.55)	0.962
	other	12.08 (7.04)	938	12.65 (7.51)	775	0.08 (-0.02 to 0.17)	0.31 (-0.28 to 0.91)	
Social-emotional-behavioural functioning baseline ‡	*normal*	11.51 (6.03)	2,531	11.59 (9.28)	2,660	0.01 (-0.04 to 0.07)	0.10 (-0.29 to 0.49)	0.150
	borderline	15.94 (6.95)	504	16.15 (6.38)	484	0.03 (-0.09 to 0.16)	0.21 (-0.61 to 1.03)	
	abnormal	17.97 (7.06)	458	19.13 (6.06)	469	0.18 (0.05 to 0.30)	1.07 (0.29 to 1.84)	
**School broad context**								
Urbanity	*Urban*	13.02 (8.24)	3,020	13.41 (9.96)	3,066	0.04 (-0.01 to 0.09)	0.26 (-0.13 to 0.65)	0.981
	Rural	12.64 (8.37)	541	12.10 (9.53)	598	-0.06 (-0.18 to 0.06)	0.70 (-0.22 to 1.61)	
**School community**								
Deprivation status ‡	*<3% FSM*	11.90 (4.56)	721	13.25 (9.43)	403	0.20 (0.08 to 0.32)	0.76 (-0.12 to 1.64)	0.416
	3-22% FSM	13.12 (7.86)	2,419	13.26 (11.64)	2,800	0.01 (-0.04 to 0.07)	0.06 (-0.36 to 0.48)	
	>22% FSM	13.75 (6.36)	421	12.80 (4.07)	461	-0.18 (-0.31 to -0.05)	-0.43 (-1.30 to 0.44)	
**School operational features**								
SEL ethos ‡	*low*	13.43 (6.07)	591	13.09 (12.59)	282	-0.04 (-0.18 to 0.10)	-0.45 (-1.56 to 0.67)	0.121
	medium	13.08 (8.28)	2,118	13.08 (9.76)	2,642	0.00 (-0.06 to 0.06)	0.16 (-0.28 to 0.60)	
	high	12.33 (9.92)	852	13.28 (12.51)	740	0.09 (-0.01 to 0.18)	0.72 (-0.05 to 1.49)	

Mixed-effects linear regressions with cluster-robust maximum likelihood estimation, including schools (clusters) as random effects and adjusted for the cohort, country, school size, school sex, and the outcome at baseline. 95% CI: 95% confidence interval. p: p-value associated with the interaction term. SEL: socio-emotional learning. FMS: free school meals. Number of clusters (schools) = 84. The non-binary gender students were not included in the analysis of gender as a potential moderator due to the very small numbers. ‡ These variables were included in the models in a continuous way (they are categorised for descriptive purposes only to aid interpretation), and p-values are presented accordingly. g: Hedges' g effect size calculated as the difference in raw means between trial arms divided by the pooled standard deviation. TAU: teaching as usual. SBMT: school-based mindfulness training.

Montero-Marin J, *et al. Evid Based Ment Health* 2022; 25:117–124. doi: 10.1136/ebmental-2022-300439

Supplementary Table S7: Subgroup analyses of well-being at 1-year follow-up

Potential modifiers	Subgroup	TAU mean (SD)	N	SBMT mean (SD)	N	g (95% CI)	Adjusted mean difference (95% CI)	p-value for interaction
**Student characteristics**								
Age ‡	*11 years*	47.92 (11.26)	1,508	47.86 (14.52)	1,541	-0.01 (-0.08 to 0.07)	-0.03 (-0.89 to 0.82)	0.967
	12 years	47.35 (11.72)	1,755	47.59 (11.09)	1,821	0.02 (-0.05 to 0.09)	0.19 (-0.44 to 0.83)	
	13 years	46.93 (9.57)	303	47.12 (9.24)	316	0.02 (-0.14 to 0.18)	-0.40 (-1.79 to 0.98)	
Gender	*male*	49.68 (10.86)	1,620	50.09 (12.99)	1,550	0.03 (-0.04 to 0.10)	0.04 (-0.68 to 0.76)	0.905
	female	45.72 (12.55)	1,874	45.72 (15.01)	2,071	0.00 (-0.06 to 0.06)	0.09 (-0.66 to 0.84)	
Ethnicity	*white*	47.27 (12.10)	2,544	47.39 (14.37)	2,833	0.01 (-0.05 to 0.06)	-0.02 (-0.67 to 0.63)	0.212
	other	48.13 (12.55)	938	48.47 (8.37)	779	0.03 (-0.06 to 0.13)	0.47 (-0.42 to 1.35)	
Well-being baseline ‡	*low*	41.03 (9.55)	494	40.60 (11.11)	515	-0.04 (-0.17 to 0.08)	-0.81 (-2.14 to 0.53)	0.420
	medium	47.45 (9.95)	2,479	47.66 (11.56)	2,530	0.02 (-0.04 to 0.08)	0.17 (-0.44 to 0.78)	
	high	53.52 (10.97)	569	53.47 (10.95)	620	-0.01 (-0.12 to 0.11)	-0.03 (-1.31 to 1.25)	
**School broad context**								
Urbanity	*Urban*	47.52 (12.65)	3,027	47.45 (12.76)	3,077	-0.01 (-0.06 to 0.05)	-0.02 (-0.59 to 0.55)	0.916
	Rural	47.84 (19.04)	539	48.73 (17.65)	601	0.05 (-0.07 to 0.17)	-0.59 (-3.44 to 2.25)	
**School community**								
Deprivation status ‡	*<3% FSM*	48.69 (15.28)	719	47.29 (18.13)	406	-0.09 (-0.21 to 0.04)	-1.17 (-2.95 to 0.61)	0.075
	3-22% FSM	47.25 (12.80)	2,425	47.57 (15.36)	2,808	0.02 (-0.03 to 0.08)	0.15 (-0.52 to 0.82)	
	>22% FSM	47.55 (10.27)	422	48.53 (5.60)	464	0.12 (-0.01 to 0.25)	0.58 (-0.69 to 1.84)	
**School operational features**								
SEL ethos ‡	*low*	47.45 (15.32)	592	47.35 (10.48)	286	-0.01 (-0.15 to 0.13)	-0.41 (-3.08 to 2.25)	0.703
	medium	47.48 (12.89)	2,122	47.86 (14.92)	2,650	0.03 (-0.03 to 0.08)	0.25 (-0.44 to 0.94)	
	high	47.88 (14.01)	852	47.08 (13.89)	742	-0.06 (-0.16 to 0.04)	-0.86 (-1.78 to 0.06)	

Mixed-effects linear regressions with cluster-robust maximum likelihood estimation, including schools (clusters) as random effects and adjusted for the cohort, country, school size, school sex, and the outcome at baseline. 95% CI: 95% confidence interval. p: p-value associated with the interaction term. SEL: socio-emotional learning. FMS: free school meals. Number of clusters (schools) = 84. The non-binary gender students were not included in the analysis of gender as a potential moderator due to the very small numbers. ‡ These variables were included in the models in a continuous way (they are categorised for descriptive purposes only to aid interpretation), and p-values are presented accordingly. g: Hedges' g effect size calculated as the difference in raw means between trial arms divided by the pooled standard deviation. TAU: teaching as usual. SBMT: school-based mindfulness training.

Montero-Marin J, *et al. Evid Based Ment Health* 2022; 25:117–124. doi: 10.1136/ebmental-2022-300439

Supplementary Table S8: Subgroup analyses of risk for depression at post-intervention

Potential modifiers	Subgroup	TAU mean (SD)	N	SBMT mean (SD)	N	g (95% CI)	Adjusted mean difference (95% CI)	p-value for interaction
**Student characteristics**								
Age ‡	*11 years*	15.04 (8.80)	1,601	15.61 (11.50)	1,575	0.06 (-0.01 to 0.13)	0.91 (0.07 to 1.76)	0.052
	12 years	15.57 (11.63)	1,857	15.52 (12.12)	1,875	0.00 (-0.07 to 0.06)	0.10 (-0.60 to 0.79)	
	13 years	16.78 (11.89)	335	16.08 (9.27)	318	-0.07 (-0.22 to 0.09)	-0.61 (-2.26 to 1.03)	
Gender	*male*	13.45 (10.44)	1,747	13.40 (15.02)	1,564	-0.01 (-0.07 to 0.06)	0.09 (-0.62 to 0.80)	0.610
	female	19.04 (16.43)	1,973	19.35 (21.29)	2,143	0.02 (-0.05 to 0.08)	0.36 (-0.57 to 1.29)	
Ethnicity	*white*	16.58 (15.64)	2,719	16.85 (17.21)	2,892	0.02 (-0.04 to 0.07)	0.23 (-0.45 to 0.92)	0.982
	other	16.01 (15.70)	986	16.59 (14.18)	805	0.04 (-0.06 to 0.13)	0.31 (-0.80 to 1.41)	
Risk for depression baseline ‡	*low*	13.46 (10.73)	2,612	13.42 (11.10)	2,546	-0.01 (-1.73 to 1.72)	0.01 (-0.57 to 0.58)	0.218
	at risk	20.70 (11.19)	824	22.05 (13.79)	861	0.11 (0.01 to 0.20)	1.29 (0.04 to 2.55)	
	case	28.39 (18.78)	353	28.47 (12.69)	359	0.01 (-0.14 to 0.15)	0.62 (-1.26 to 2.49)	
**School broad context**								
Urbanity	*Urban*	16.50 (14.72)	3,205	17.14 (17.46)	3,173	0.04 (-0.01 to 0.09)	0.46 (-0.21 to 1.13)	0.443
	Rural	16.05 (19.88)	588	15.11 (13.66)	595	-0.06 (-0.17 to 0.06)	-0.47 (-2.19 to 1.24)	
**School community**								
Deprivation status ‡	*<3% FSM*	15.05 (13.85)	738	16.82 (13.11)	407	0.13 (0.01 to 0.25)	0.89 (-0.52 to 2.30)	0.133
	3-22% FSM	16.81 (15.28)	2,596	17.10 (19.30)	2,877	0.02 (-0.04 to 0.07)	0.29 (-0.41 to 0.99)	
	>22% FSM	16.46 (21.42)	459	15.09 (11.44)	484	-0.08 (-0.21 to 0.05)	-0.70 (-2.01 to 0.61)	
**School operational features**								
SEL ethos ‡	*low*	16.72 (11.82)	661	17.02 (19.39)	300	0.02 (-0.12 to 0.16)	-0.07 (-1.83 to 1.69)	0.477
	medium	16.54 (16.17)	2,264	16.78 (17.14)	2,699	0.01 (-0.04 to 0.07)	0.25 (-0.53 to 1.03)	
	high	15.92 (18.26)	868	16.80 (23.01)	769	0.04 (-0.05 to 0.14)	0.86 (-0.54 to 2.26)	

Mixed-effects linear regressions with cluster-robust maximum likelihood estimation, including schools (clusters) as random effects and adjusted for the cohort, country, school size, school sex, and the outcome at baseline. 95% CI: 95% confidence interval. p: p-value associated with the interaction term. SEL: socio-emotional learning. FMS: free school meals. Number of clusters (schools) = 84. The non-binary gender students were not included in the analysis of gender as a potential moderator due to the very small numbers. ‡ These variables were included in the models in a continuous way (they are categorised for descriptive purposes only to aid interpretation), and p-values are presented accordingly. g: Hedges' g effect size calculated as the difference in raw means between trial arms divided by the pooled standard deviation. TAU: teaching as usual. SBMT: school-based mindfulness training.

Montero-Marin J, *et al. Evid Based Ment Health* 2022; 25:117–124. doi: 10.1136/ebmental-2022-300439

Supplementary Table S9: Subgroup analyses of social-emotional-behavioural functioning at post-intervention

Potential modifiers	Subgroup	TAU mean (SD)	N	SBMT mean (SD)	N	g (95% CI)	Adjusted mean difference (95% CI)	p-value for interaction
**Student characteristics**								
Age ‡	*11 years*	12.89 (9.59)	1,598	13.49 (10.28)	1,565	0.06 (-0.01 to 0.13)	0.45 (-0.07 to 0.97)	0.234
	12 years	13.27 (9.05)	1,857	13.28 (8.65)	1,871	0.00 (-0.06 to 0.07)	0.06 (-0.37 to 0.48)	
	13 years	13.52 (7.32)	335	13.09 (7.99)	316	-0.06 (-0.21 to 0.10)	0.12 (-0.78 to 1.02)	
Gender	*male*	12.10 (8.35)	1,745	12.13 (9.86)	1,557	0.00 (-0.07 to 0.07)	0.11 (-0.37 to 0.59)	0.724
	female	14.00 (10.21)	1,972	14.26 (12.01)	2,135	0.02 (-0.04 to 0.08)	0.23 (-0.23 to 0.69)	
Ethnicity	*white*	13.43 (8.86)	2,717	13.51 (10.73)	2,880	0.01 (-0.04 to 0.06)	0.23 (-0.19 to 0.65)	0.633
	other	12.30 (7.84)	985	12.75 (8.21)	802	0.06 (-0.04 to 0.15)	0.16 (-0.36 to 0.67)	
Social-emotional-behavioural functioning baseline ‡	*normal*	11.45 (6.73)	2,681	11.61 (8.83)	2,701	0.02 (-0.03 to 0.07)	0.20 (-0.17 to 0.57)	0.220
	borderline	16.26 (6.81)	553	16.42 (6.24)	498	0.03 (-0.10 to 0.15)	0.16 (-0.59 to 0.92)	
	abnormal	18.84 (6.39)	486	19.64 (6.24)	498	0.13 (0.00 to 0.25)	0.76 (0.03 to 1.50)	
**School broad context**								
Urbanity	*Urban*	13.19 (10.18)	3,203	13.56 (11.79)	3,156	0.03 (-0.02 to 0.08)	0.23 (-0.17 to 0.63)	0.732
	Rural	12.72 (9.93)	587	12.34 (7.32)	596	-0.04 (-0.16 to 0.07)	0.49 (-0.43 to 1.42)	
**School community**								
Deprivation status ‡	*<3% FSM*	11.85 (4.61)	736	13.45 (6.45)	407	0.30 (0.18 to 0.42)	1.04 (0.30 to 1.78)	0.150
	3-22% FSM	13.34 (9.67)	2,595	13.48 (12.84)	2,865	0.01 (-0.04 to 0.07)	0.08 (-0.35 to 0.51)	
	>22% FSM	13.79 (4.92)	459	12.62 (4.60)	480	-0.25 (-0.37 to -0.12)	-0.53 (-1.32 to 0.26)	
**School operational features**								
SEL ethos ‡	*low*	13.52 (5.14)	662	13.41 (13.93)	296	-0.01 (-0.15 to 0.13)	-0.68 (-1.54 to 0.18)	0.082
	medium	13.24 (10.93)	2,261	13.34 (10.88)	2,688	0.01 (-1.76 to 1.78)	0.19 (-0.27 to 0.66)	
	high	12.49 (11.48)	867	13.39 (13.85)	768	0.07 (-0.03 to 0.17)	0.71 (-0.04 to 1.46)	

Mixed-effects linear regressions with cluster-robust maximum likelihood estimation, including schools (clusters) as random effects and adjusted for the cohort, country, school size, school sex, and the outcome at baseline. 95% CI: 95% confidence interval. p: p-value associated with the interaction term. SEL: socio-emotional learning. FMS: free school meals. Number of clusters (schools) = 84. The non-binary gender students were not included in the analysis of gender as a potential moderator due to the very small numbers. ‡ These variables were included in the models in a continuous way (they are categorised for descriptive purposes only to aid interpretation), and p-values are presented accordingly. g: Hedges' g effect size calculated as the difference in raw means between trial arms divided by the pooled standard deviation. TAU: teaching as usual. SBMT: school-based mindfulness training.

Supplementary Table S10: Subgroup analyses of well-being at post-intervention

Potential modifiers	Subgroup	TAU mean (SD)	N	SBMT mean (SD)	N	g (95% CI)	Adjusted mean difference (95% CI)	p-value for interaction
**Student characteristics**								
Age ‡	*11 years*	48.43 (12.00)	1,601	47.89 (11.12)	1,580	-0.05 (-0.12 to 0.02)	-0.53 (-1.25 to 0.20)	0.249
	12 years	47.94 (12.07)	1,860	47.84 (10.83)	1,878	-0.01 (-0.07 to 0.06)	-0.17 (-0.82 to 0.47)	
	13 years	46.82 (8.61)	336	47.59 (10.68)	317	0.08 (-0.07 to 0.23)	0.27 (-1.00 to 1.55)	
Gender	*male*	50.16 (12.96)	1,749	50.11 (11.88)	1,569	0.00 (-0.07 to 0.06)	-0.37 (-1.07 to 0.34)	0.537
	female	46.24 (13.77)	1,975	46.12 (15.28)	2,145	-0.01 (-0.07 to 0.05)	-0.14 (-0.86 to 0.58)	
Ethnicity	*white*	47.97 (12.00)	2,722	47.81 (13.46)	2,899	-0.01 (-0.07 to 0.04)	-0.26 (-0.83 to 0.30)	0.608
	other	48.32 (14.76)	987	47.95 (9.08)	805	-0.03 (-0.12 to 0.06)	-0.14 (-1.08 to 0.81)	
Well-being baseline ‡	*low*	41.02 (9.27)	538	40.00 (12.46)	533	-0.09 (-0.21 to 0.03)	-1.22 (-2.59 to 0.16)	0.493
	medium	48.01 (9.72)	2,621	47.88 (8.65)	2,592	-0.01 (-0.07 to 0.04)	-0.11 (-0.62 to 0.40)	
	high	54.32 (8.67)	614	54.16 (7.79)	633	-0.02 (-0.13 to 0.09)	-0.21 (-1.18 to 0.76)	
**School broad context**								
Urbanity	*Urban*	48.05 (13.59)	3,209	47.61 (13.53)	3,179	-0.03 (-0.08 to 0.02)	-0.31 (-0.89 to 0.27)	0.863
	Rural	48.05 (16.34)	588	49.01 (9.52)	596	0.07 (-0.04 to 0.19)	-0.25 (-1.58 to 1.07)	
**School community**								
Deprivation status ‡	*<3% FSM*	48.72 (13.58)	738	47.49 (5.85)	407	-0.11 (-0.23 to 0.01)	-0.69 (-2.08 to 0.71)	0.110
	3-22% FSM	47.75 (13.76)	2,598	47.58 (15.03)	2,884	-0.01 (-0.07 to 0.04)	-0.34 (-0.96 to 0.28)	
	>22% FSM	48.56 (11.59)	461	49.64 (4.40)	484	0.12 (0.00 to 0.25)	0.71 (-0.49 to 1.90)	
**School operational features**								
SEL ethos ‡	*low*	48.13 (14.67)	663	48.28 (17.37)	302	0.01 (-0.13 to 0.15)	-0.04 (-2.36 to 2.28)	0.931
	medium	48.04 (14.28)	2,267	47.79 (13.51)	2,703	-0.02 (-0.07 to 0.04)	-0.38 (-1.05 to 0.29)	
	high	48.00 (13.83)	867	47.80 (14.15)	770	-0.01 (-0.11 to 0.08)	-0.19 (-1.01 to 0.63)	

Mixed-effects linear regressions with cluster-robust maximum likelihood estimation, including schools (clusters) as random effects and adjusted for the cohort, country, school size, school sex, and the outcome at baseline. 95% CI: 95% confidence interval. p: p-value associated with the interaction term. SEL: socio-emotional learning. FMS: free school meals. Number of clusters (schools) = 84. The non-binary gender students were not included in the analysis of gender as a potential moderator due to the very small numbers. ‡ These variables were included in the models in a continuous way (they are categorised for descriptive purposes only to aid interpretation), and p-values are presented accordingly. g: Hedges' g effect size calculated as the difference in raw means between trial arms divided by the pooled standard deviation. TAU: teaching as usual. SBMT: school-based mindfulness training.

Supplement E: Model selection, latent moderator interpretation, and student classification according to the LPA

The baseline student characteristics that were used to develop the LPA, for the sample available at each time point, are presented below (*Table a*). A random 50% split of the total sample resulted in two sub-samples of n_1 = 4,154 students (test sample) and n_2 = 4,222 students (validation sample), both with 84 schools (see *Table b* below). Using LPA to define a baseline latent moderator, models with 1 through 8 profiles were compared in the test sample first. All models were well identified. As can be observed in *Table c* below, more latent profiles resulted in lower values for the information criteria presented, and hence suggested a better model fit. However, the LMR-LRT identified only three profiles, and the best entropy value was obtained with two profiles. In addition, the Elbow Plot (*Figure a* below) showed the steepest slope with only two profiles. Given all this information, we compared the two- and three-profile models for conceptual interpretability and clarity. The three-profile model replicated the higher risk profile of the two-profile model, and largest profile was split into two non-risk profiles. For a better balance between fit and parsimony, and because the additional profile did not contribute to the interpretation of the sample, we chose the two-profile model. To validate the structure of the selected two-profile model, we repeated the process with the validation sample (see *Table c* and *Figure a* below), in which all defining characteristics of the latent classes were replicated, supporting the validity of our two-profile model. Following confirmation of a two-profile model structure from the two independent split-half samples, the dataset was recombined, and the same method of LPA was applied on the full sample (see *Table c* and *Figure a* below). This allowed us to estimate the latent profile measurement model, generating weights that reflect individual profile membership, as well as the measurement error of the latent profile variable. Then, the latent profile variable was used to estimate the subsequent auxiliary model (i.e., moderation for a linear cluster-robust regression), conditional on the latent profile. Subsequent analyses were developed using the total sample.

The largest subgroup of students (72.8%) was mainly characterised by lower values of risk for depression and social-emotional-behavioural functioning, as well as higher values of well-being. This subgroup was also younger, more often identified as males, and other ethnic backgrounds than ‘whites’, had a higher SEL ethos, and were more often from rural areas (see *Table d* below). Students in this subgroup were much less likely to be at risk of suffering from mental health problems, and thus, this sub-group was labelled as “low risk”. On the contrary, the other subgroup of students (27.2%) had higher values of risk for depression and social-emotional-behavioural functioning, as well as lower values of well-being, and were older, more often identified as females and ‘whites’, had a lower SEL ethos, and were more often from urban areas (see *Table d* below). Students in this subgroup were more likely to be at risk of suffering from mental health problems, and thus, this subgroup was labelled as “high risk”. The mean values of the “low risk” subgroup were in the low category of risk for depression and social-emotional-behavioural functioning, and in the medium category of well-being; while the mean values of the “high risk” subgroup were in the at-risk category of risk for depression and social-emotional-behavioural functioning, and in the low category of well-being (for more information regarding the cut-offs of this measures see the Supplement B). This two-subgroup model, that was used to assign students into latent profiles, was characterised by high posterior probabilities for all latent profiles across both the total sample and the randomly selected subsamples, suggesting low classification error (see *Table e*).

Table a: Baseline student characteristics used to develop the latent profile analysis for the sample available at each time point regarding the primary outcomes.

Variables	T0 (N = 8,376)	T1 (N = 8,072)	T2 (N = 7,588)	T3 (N = 7,263)
Student				
Age, mean (SD)	12.16 (0.57)	12.16 (0.57)	12.16 (0.57)	12.16 (0.57)
Gender, female, n (%)	4,509 (54.9%)	3,389 (55.3%)	3,181 (55.4%)	3,044 (55.4%)
Ethnicity, white, n (%)	6,202 (75.7%)	5,967 (75.7%)	5,631 (75.8%)	5,384 (75.7%)
CESD, mean (SD)	13.45 (9.89)	13,37 (9.84)	13.19 (9.75)	13.14 (9.72)
SDQ, mean (SD)	11.78 (6.46)	11,70 (6.44)	11.61 (6.41)	11.52 (6.39)
WEMWBS, mean (SD)	49.68 (9.73)	49.73 (9.71)	49.88 (9.67)	49.91 (9.63)
School				
Urbanity, urban, n (%)	7,089 (84.6%)	6,831 (84.6%)	6,402 (84.4%)	6,119 (84.2%)
Deprivation (% free school meals), mean (SD)	11.99 (8.82)	11.92 (8.84)	11.73 (8.70)	11.69 (8.80)
SEL ethos, mean (SD)	50.30 (9.89)	50.43 (9.86)	50.68 (9.91)	50.81 (9.91)

T0: Baseline. T1: Pre-intervention. T2: Post-intervention. T3: 1-year follow-up. (%). Age was not fixed (similarities are due to the small age range). CESD: Center for Epidemiological Studies Depression. SDQ: Strengths and Difficulties Questionnaire. WEMWBS: Warwick-Edinburgh Mental Well-being Scale. SEL ethos: Social and Emotional Learning ethos. Values are means (SD) or frequencies (percentages).

Montero-Marin J, *et al. Evid Based Ment Health* 2022; 25:117–124. doi: 10.1136/ebmental-2022-300439

Table b: Baseline student characteristics in the test, validation, and total samples

Variables	Test (N = 4,154)	Validation (N = 4,222)	Total (N = 8,376)
Student			
Age, mean (SD)	12.16 (0.57)	12.16 (0.57)	12.16 (0.57)
Gender, female, n (%)	2,355 (56.7%)	2,335 (55.3%)	4,509 (54.9%)
Ethnicity, white, n (%)	3,506 (76.4%)	3,167 (75.0%)	6,202 (75.7%)
SDQ, mean (SD)	11.84 (6.47)	11.71 (6.45)	11.78 (6.46)
CESD, mean (SD)	13.60 (9.98)	13.31 (9.79)	13.45 (9.89)
WEMWBS, mean (SD)	49.65 (9.78)	49.70 (9.68)	49.68 (9.73)
School			
Urbanity, urban, n (%)	3,506 (84.4%)	3,585 (84.9%)	7,089 (84.6%)
Deprivation (% free school meals), mean (SD)	11.78 (8.78)	12.19 (8.86)	11.99 (8.82)
SEL ethos, mean (SD)	50.33 (9.93)	50.27 (9.85)	50.30 (9.89)

SDQ: Strengths and Difficulties Questionnaire. CESD: Center for Epidemiological Studies Depression.

WEMWBS: Warwick-Edinburgh Mental Well-being Scale. SEL ethos: Social and Emotional Learning ethos.

Values are means (SD) or frequencies (%).

Montero-Marin J, *et al. Evid Based Ment Health* 2022; 25:117–124. doi: 10.1136/ebmental-2022-300439

Table c: Fit indices for models containing 1 to 8 latent profiles in the test, validation, and total samples

Latent profiles	AIC	CAIC	BIC	sBIC	LL	Npar	LMR-LRT	BLRT	Entropy
Test sample (N = 4,154)									
1	169746.51	169856.48	169841.48	169793.82	-84858.25	15	-	-	-
2	165152.07	165335.36	165310.36	165230.92	-82551.03	25	4559.71 (<.001)	<.001	0.86
3	163548.54	163805.15	163770.15	163658.94	-81739.27	35	1604.27 (.004)	<.001	0.81
4	162785.96	163115.89	163070.89	162927.90	-81347.98	45	773.30 (.697)	<.001	0.80
5	162160.04	162533.29	162508.29	162333.52	-81025.02	55	638.26 (.253)	<.001	0.80
6	161836.12	162312.69	162247.69	162041.15	-80853.06	65	339.84 (.785)	<.001	0.79
7	161422.17	161972.06	161897.06	161658.74	-80636.09	75	428.80 (.642)	<.001	0.82
8	161004.57	161627.78	161542.78	161272.69	-80417.29	85	432.41 (.610)	<.001	0.81
Validation sample (N = 4,222)									
Latent profiles	AIC	CAIC	BIC	sBIC	LL	Npar	LMR-LRT	BLRT	Entropy
1	172207.30	172317.52	172302.52	172254.86	-86088.65	15	-	-	-
2	167638.22	167821.92	167796.92	167717.48	-83794.11	25	4534.77 (<.001)	<.001	0.85
3	165925.27	166182.46	166147.46	166036.24	-82927.64	35	1712.43 (.007)	<.001	0.84
4	165173.16	165360.84	165315.84	165315.84	-82541.58	45	762.97 (.633)	<.001	0.83
5	164598.81	165002.96	164947.96	164773.19	-82244.41	55	587.32 (.410)	<.001	0.79
6	164287.81	164765.43	164700.43	164493.89	-82078.90	65	327.08 (.764)	<.001	0.78
7	163874.92	164426.02	164351.02	164112.71	-81862.46	75	416.25 (.287)	<.001	0.80
8	163498.65	164123.24	164038.24	163768.15	-81664.33	85	391.56 (.577)	<.001	0.81
Total sample (N = 8,376)									
Latent profiles	AIC	CAIC	BIC	sBIC	LL	Npar	LMR-LRT	BLRT	Entropy
1	341939.11	342059.60	342044.60	341996.94	-170954.55	15	-	-	-
2	332770.43	332971.26	332946.26	332866.81	-166360.22	25	9088.07 (<.001)	<.001	0.85
3	329464.88	329746.04	329711.04	329599.82	-164697.44	35	3289.14 (<.001)	<.001	0.83
4	327945.73	328107.22	328262.22	328119.22	-163927.86	45	1522.30 (.667)	<.001	0.82
5	326710.10	327151.92	327096.92	326922.14	-163300.05	55	1241.88 (.277)	<.001	0.79
6	326072.81	326594.96	326529.96	326323.40	-162971.40	65	650.09 (.793)	<.001	0.78
7	325267.56	325870.04	325795.04	325556.70	-162558.78	75	816.22 (.336)	<.001	0.80
8	324449.84	325132.66	325047.66	324777.54	-162139.92	85	828.53 (.728)	<.001	0.81

AIC = Akaike information criterion. CAIC = consistent Akaike information criterion. BIC = Bayesian information criterion. sBIC = sample-size-adjusted BIC. LL = Loglikelihood value. Npar = Number of free parameters. LMR-LRT = Lo–Mendell–Rubin adjusted likelihood ratio test. BLRT = bootstrapped likelihood ratio test. Entropy = index of classification accuracy.

Figure a: Elbow plot for LPA involving the test, validation, and total samples

Test sample (N = 4,154)

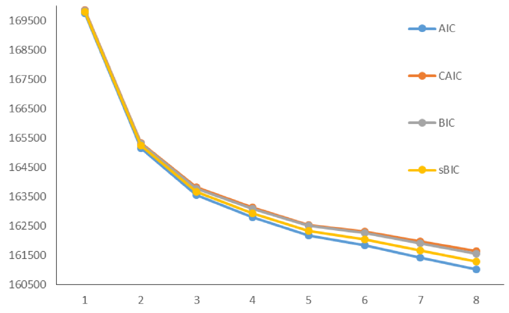

Validation sample (N = 4,222)

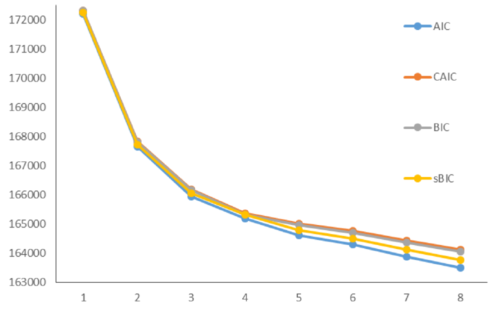

Total sample (N = 8,376)

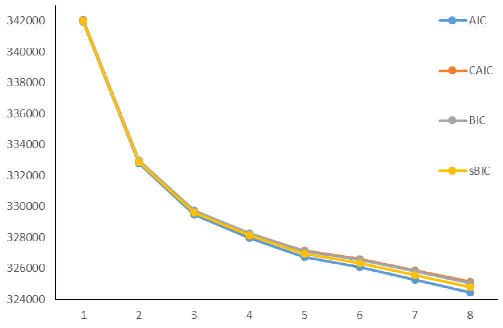

AIC = Akaike information criterion. CAIC = consistent Akaike information criterion.
BIC = Bayesian information criterion. sBIC = sample-size-adjusted BIC.

Montero-Marin J, *et al. Evid Based Ment Health* 2022; 25:117–124. doi: 10.1136/ebmental-2022-300439

Table d: Latent profiles and associated student characteristics in the test, validation, and total samples

Test sample (N = 4,154)		
Variables	LP I (72.8%): *Low Risk Profile* (N = 3,024)	LP II (27.2%): *High Risk Profile* (N = 1,130)
M (SE)		
Age	12.15 (0.03)	12.20 (0.03)
SDQ	9.11 (0.16)	19.21 (0.25)
CESD	8.95 (0.15)	26.06 (0.50)
WEMWBS	53.53 (0.22)	39.22 (0.32)
Deprivation (% free school meals)	11.64 (1.00)	12.17 (0.88)
SEL ethos	50.58 (1.15)	49.67 (1.18)
N (%)		
Gender, female	1,645 (54.4%)	712 (63.1%)
Ethnicity, white	2,301 (76.1%)	874 (77.4%)
Urbanity, urban	2,525 (83.5 %)	979 (86.7 %)
Validation sample (N = 4,222)		
Variables	LP I (72.0%): *Low Risk Profile* (N = 3,037)	LP II (28.0%): *High Risk Profile* (N = 1,185)
M (SE)		
Age	12.14 (0.03)	12.23 (0.03)
SDQ	8.87 (0.15)	18.99 (0.25)
CESD	8.66 (0.17)	25.21 (0.54)
WEMWBS	53.55 (0.20)	39.85 (0.39)
Deprivation (% free school meals)	12.01 (0.99)	12.27 (0.91)
SEL ethos	50.32 (1.10)	50.14 (1.19)
N (%)		
Gender, female	1,607 (52.9%)	728 (61.4%)
Ethnicity, white	2,250 (74.1%)	914 (77.1%)
Urbanity, urban	2,572 (84.7 %)	1,013 (85.5 %)
Total sample (N = 8,376)		
Variables	LP I (72.8%): *Low Risk Profile* (N = 6,101)	LP II (27.2%): *High Risk Profile* (N = 2,275)
M (SE)		
Age	12.14 (0.03)	12.21 (0.03)
SDQ	8.99 (0.13)	19.10 (0.18)
CESD	8.81 (0.13)	25.63 (0.37)
WEMWBS	53.54 (0.17)	39.53 (0.25)
Deprivation (% free school meals)	11.95 (0.99)	12.09 (0.87)
SEL ethos	50.45 (1.11)	49.91 (1.16)
N (%)		
Gender, female	3,264 (53.5%)	1,426 (62.7%)
Ethnicity, white	4,582 (75.1%)	1,761 (77.4%)
Urbanity, urban	5,125 (84.0%)	1,961 (86.2%)

Profile counts and proportions are based on estimated posterior probabilities. SDQ: Strengths and Difficulties Questionnaire. CESD: Center for Epidemiological Studies Depression. WEMWBS: Warwick-Edinburgh Mental Well-being Scale. SEL ethos: Social and Emotional Learning ethos. Values are means (SE) or frequencies (%).

Montero-Marin J, *et al. Evid Based Ment Health* 2022; 25:117–124. doi: 10.1136/ebmental-2022-300439

Table e: Classification probabilities for the most likely latent class membership (row) by latent class (column)

Test sample (N = 4,154)		
Latent Profiles	I	II
I	.98	02
II	.08	.92
Validation sample (N = 4,222)		
Latent Profiles	I	II
I	.97	03
II	.08	.92
Total sample (N = 8,376)		
Latent Profiles	I	II
I	.97	.03
II	.09	.91

Latent Profile I: low risk. Latent Profile II: high risk.

Montero-Marin J, *et al. Evid Based Ment Health* 2022; 25:117–124. doi: 10.1136/ebmental-2022-300439

Supplementary Figure S1: Graphical representation of the distribution of predictor variables between latent profiles

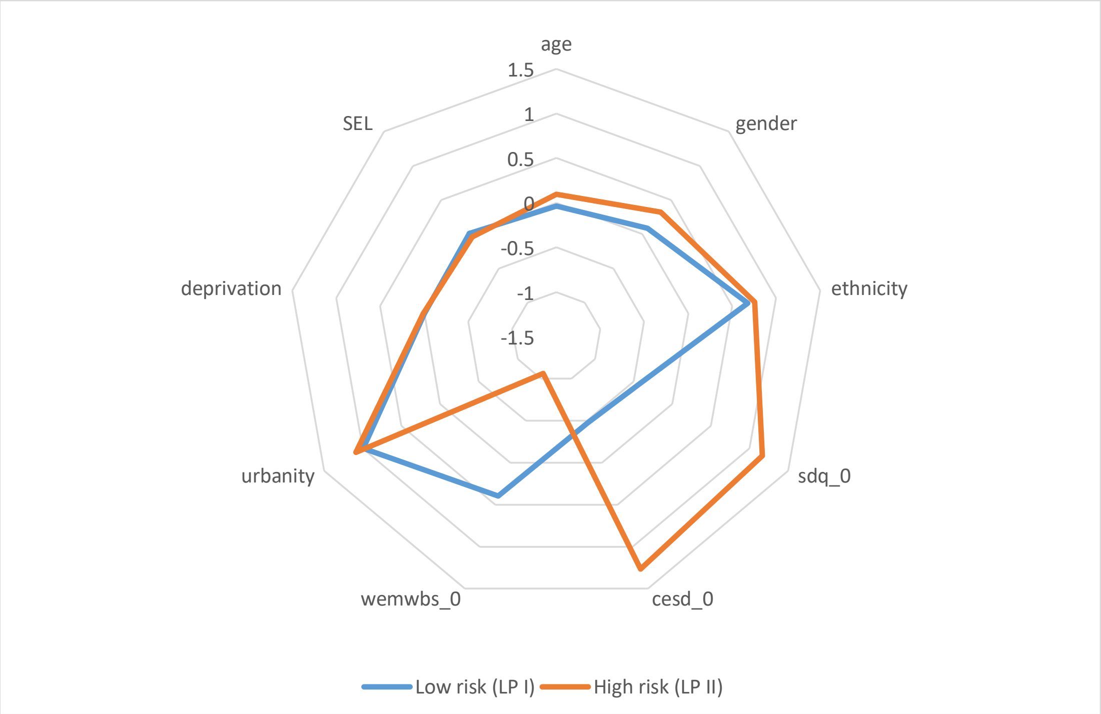

Total sample N = 8,376. Due to the different scaling of the continuous and dichotomous items included in the latent profile analysis, all mean scores and proportions for each predictor were standardised and z-scores were used to present the distribution between mean scores and proportions for each profile. SDQ: Strengths and Difficulties Questionnaire. CESD: Center for Epidemiological Studies Depression. WEMWBS: Warwick-Edinburgh Mental Well-being Scale. SEL ethos: Social and Emotional Learning ethos.

Montero-Marin J, *et al. Evid Based Ment Health* 2022; 25:117–124. doi: 10.1136/ebmental-2022-300439

Supplementary Table S11: Latent Profiles and Associated Baseline Characteristics by trial arm

Variables	LP I (low risk)		LP II (high risk)	
	TAU (N = 3,056)	SBMT (N = 3,045)	TAU (N = 1,176)	SBMT (N = 1,099)
Student				
Age, mean (SD)	12.15 (0.56)	12.14 (0.57)	12.20 (0.58)	12.24 (0.58)
Gender, female, n (%)	1,632 (55.1%)	1,531 (52.0%)	718 (64.6%)	628 (60.7%)
Ethnicity, white, n (%)				
SDQ, mean (SD)	8.91 (4.54)	9.05 (4.51)	19.36 (4.61)	19.23 (4.61)
CESD, mean (SD)	8.75 (5.07)	8.80 (5.00)	26.10 (8.51)	25.87 (8.93)
WEMWBS, mean (SD)	53.62 (7.34)	53.47 (7.29)	39.54 (7.60)	39.05 (7.57)
School				
Urbanity, urban, n (%)	2,541 (83.1%)	2,586 (84.9%)	1,041 (88.5%)	921 (83.8%)
Deprivation (% free school meals), mean (SD)	13.01 (7.86)	10.89 (9.90)	12.68 (7.33)	11.46 (9.24)
SEL ethos, mean (SD)	50.24 (9.34)	50.66 (10.51)	49.84 (9.93)	49.96 (9.57)

LP: latent profile. TAU: teaching as usual. SBMT: school-based mindfulness training. SDQ: Strengths and Difficulties Questionnaire. CESD: Center for Epidemiological Studies Depression. WEMWBS: Warwick-Edinburgh Mental Well-being Scale. SEL ethos: Social and Emotional Learning ethos. Values are means (SD) or frequencies (%).

Supplementary Figure S2: Risk for depression, social-emotional-behavioural functioning, and well-being at each time point (baseline, pre-intervention, post-intervention, 1-year follow-up) by latent profile and group

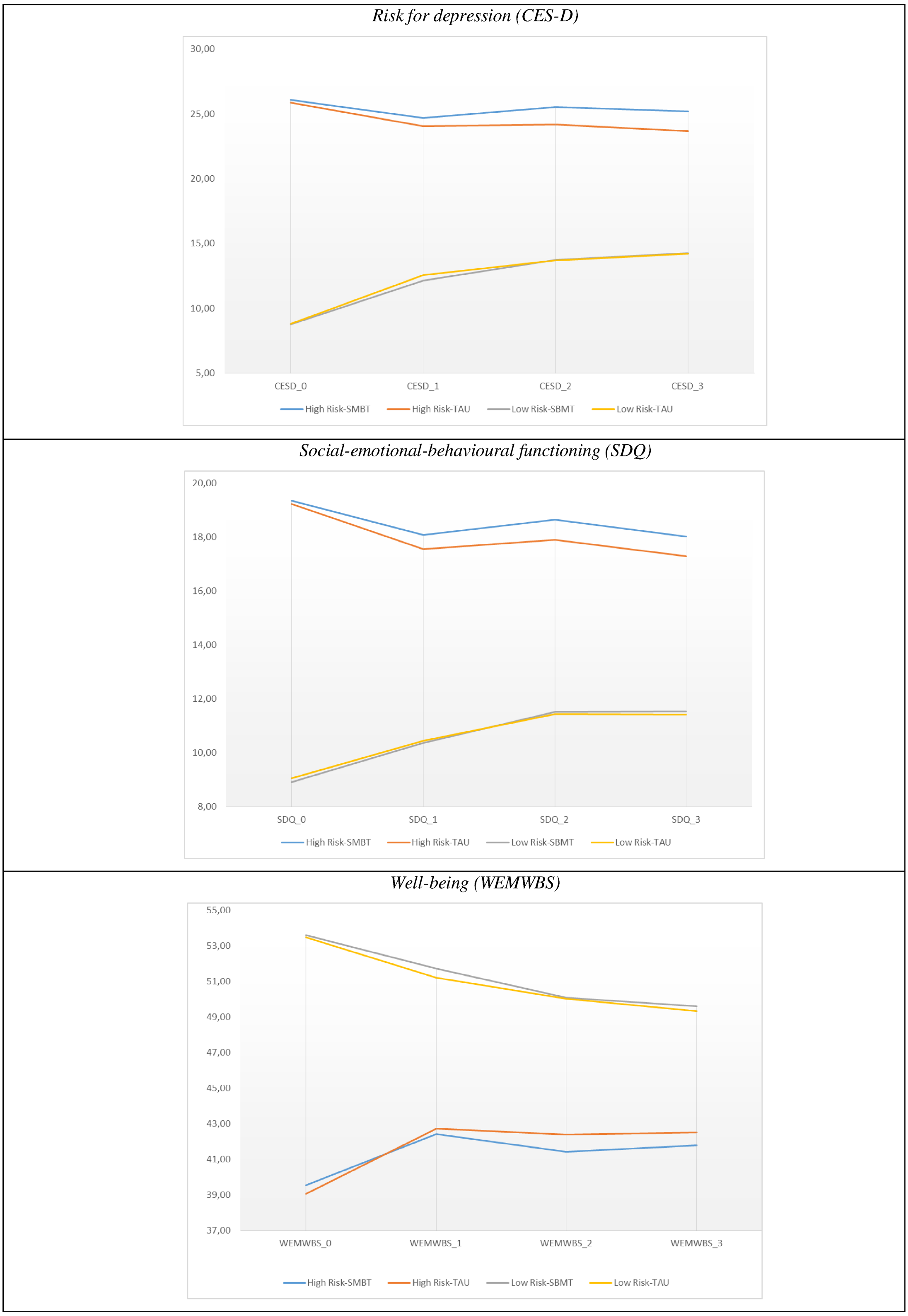

Montero-Marin J, *et al. Evid Based Ment Health* 2022; 25:117–124. doi: 10.1136/ebmental-2022-300439

Supplementary Table S12: Subgroup analyses of primary outcomes at post-intervention and 1-year follow-up by latent profile (high/low risk) moderator

Timepoint/Outcome	Latent profile	TAU mean (SD)	N	SBMT mean (SD)	N	g (95% CI)	Adjusted mean difference (95% CI)	p-value for interaction
**1-year follow-up**								
Risk for depression	High risk	23.69 (12.49)	910	25.18 (12.03)	949	0.12 (0.03 to 0.21)	1.47 (0.37 to 2.57)	0.016
	Low risk	14.23 (10.60)	2,656	14.27 (10.46)	2,723	0.00 (-0.05 to 0.06)	-0.13 (-0.85 to 0.59)	
Social-emotional-behavioural functioning	High risk	17.30 (6.41)	909	18.03 (6.17)	947	0.12 (0.03 to 0.21)	0.57 (0.02 to 1.13)	0.209
	Low risk	11.42 (6.28)	2,652	11.54 (6.20)	2,717	0.01 (-0.03 to 0.07)	0.11 (-0.27 to 0.49)	
Well-being	High risk	42.51 (9.38)	908	41.79 (9.21)	952	-0.08 (-0.17 to -0.01)	-0.88 (-1.71 to -0.05)	0.050
	Low risk	49.32 (9.36)	2,658	49.62 (9.18)	2,726	0.03 (-0.02 to 0.09)	0.35 (-0.29 to 0.99)	
**Post-treatment**								
Risk for depression	High risk	24.19 (12.25)	1,000	25.52 (11.97)	975	0.11 (0.02 to 0.20)	1.40 (0.27 to 2.53)	0.023
	Low risk	13.71 (10.09)	2,768	13.74 (10.04)	2,818	0.00 (-0.05 to 0.06)	-0.02 (-0.61 to 0.58)	
Social-emotional-behavioural functioning	High risk	17.90 (6.32)	996	18.66 (6.16)	974	0.12 (0.03 to 0.21)	0.70 (0.17 to 1.23)	0.094
	Low risk	11.43 (6.21)	2,816	11.51 (6.06)	2,756	0.01 (-0.04 to 0.07)	0.10 (-0.30 to 0.49)	
Well-being	High risk	42.38 (9.01)	1,003	41.43 (8.97)	976	-0.11 (-0.19 to -0.02)	-1.10 (-1.98 to -0.22)	0.029
	Low risk	50.02 (8.64)	2,821	50.08 (8.73)	2,772	0.01 (-0.05 to 0.06)	0.05 (-0.47 to 0.58)	

Auxiliary model where the latent profile variable is a moderator of a mixed (hierarchical) linear regression with cluster-robust maximum likelihood estimation, including schools (clusters) as random effects and adjusted for the cohort, country, school size, school sex, and the outcome at baseline. 95% CI: 95% confidence interval. p: p-value associated with the interaction term. g: Hedges' g effect size calculated as the difference in means between trial arms divided by the pooled standard deviation. TAU: teaching as usual. SBMT: school-based mindfulness training.

Supplementary Table S13: Descriptive data for the implementation factors in the intervention arm

**Implementation factors**	**N**	**possible range**	**observed range**	**mean**	**SD**
Dose	3,265 students	0-10	0-10	8.97	2.08
Fidelity	164 classes	0-100	32-100	83.0	12.1
Quality	192 classes	1-6	2-6	3.8	0.8
Reach	35 schools	0-100	5-66	25.73	11.40
Student mindfulness practice at post-intervention	3,637 students	0-5	0-5	1.16	1.07
Student mindfulness practice at 1-year follow-up	3,598 students	0-5	0-5	0.83	0.93

N: number of students/classes/schools reporting data. Dose (number of SBMT sessions that students received): 77.2% of students in the intervention arm had a registry of the number of SBMT sessions received. Fidelity (the percentage of the total SBMT content delivered by the teacher) was rated for 82.4% (164 out of 199) of classes, while quality (teacher competency rated on the MBI-TAC-Teach) was available for 96.5% of classes (192 out of 199 classes). Reach (proportion of students receiving more than 67% of the SBMT, relative to the study year group school population) was obtained for 81.4% of schools (35 out of 43 schools). 85.9% of students reported their frequency of mindfulness practice at post-intervention, and 85.0% did so at 1-year follow-up. For further details on the measures see Supplement B above.

Supplementary Table S14: Instrumental Variable Analysis of outcomes at 1-year follow-up, with trial arm as an instrument for quality after being dichotomised

**Outcome/Implementation variables**	**N (K)**	**coefficient**	**95% CI**	**p**
CESD				
Quality: competent or more	6,139 (73)	0.55	-0.43 to 1.53	0.272
SDQ				
Quality: competent or more	6,046 (73)	0.32	-0.17 to 0.81	0.200
WEMWBS				
Quality: competent or more	6,116 (73)	-0.03	-0.87 to 0.82	0.955

Quality has been dichotomised as incompetent/beginner/advanced beginner vs competent/proficient/advanced.

N: number of students in analysis. K: number of clusters (schools) in analysis.

Supplementary Table S15: Instrumental Variable Analysis of outcomes at post-intervention, with trial arm as an instrument for the implementation variables

Implementation/Outcome variables	N (K)	coefficient	95% CI	p
CESD				
Dose	5,797 (65)	0.07	-0.01 to 0.15	0.071
Fidelity	5,968 (66)	0.003	-0.01 to 0.01	0.418
Reach	5,960 (65)	0.02	-0.01 to 0.04	0.151
Quality	6,440 (73)	0.11	-0.05 to 0.27	0.176
Practice (post-intervention)	6,424 (73)	0.45	-0.11 to 1.00	0.114
Practice (1-year follow-up)	6,192 (73)	0.46	-0.29 to 1.20	0.232
SDQ				
Dose	5,701 (65)	0.05	0.002 to 0.09	0.042
Fidelity	5,863 (66)	0.003	-0.002 to 0.01	0.212
Reach	5,858 (65)	0.01	0.0003 to 0.03	0.045
Quality	6,334 (73)	0.07	-0.03 to 0.16	0.160
Practice (post-intervention)	6,335 (73)	0.26	-0.04 to 0.57	0.092
Practice (1-year follow-up)	6,098 (73)	0.25	-0.18 to 0.67	0.256
WEMWBS				
Dose	5,770 (65)	-0.06	-0.13 to -0.002	0.043
Fidelity	5,940 (66)	-0.01	-0.01 to 0.001	0.124
Reach	5,934 (65)	-0.02	-0.04 to 0.001	0.057
Quality	6,415 (73)	-0.11	-0.25 to 0.03	0.107
Practice (post-intervention)	6,390 (73)	-0.36	-0.82 to 0.09	0.115
Practice (1-year follow-up)	6,168 (73)	-0.44	-1.08 to 0.20	0.176

N = number of students in analysis. K = number of clusters (schools) in analysis. CES-D is the "Center for Epidemiologic Studies for Depression Scale". SDQ is the "Strengths and Difficulties Questionnaire" (specifically, the Total Difficulties - self report scale was used). WEMWBS is the "Warwick-Edinburgh Mental Well-Being Scale". Dose is the number of SBMT sessions that students received. Quality is the teacher competency delivering the SBMT independently evaluated by using the Mindfulness-based Interventions Teaching Assessment Criteria (MBI-TAC). Fidelity is the independently rated percentage of the total original SBMT content delivered by the teacher. Reach is the percentage of school study students receiving ≥ 67% of SBMT sessions. Practice is the amount of home-based student mindfulness practice during/after the intervention. 95% CI: 95% confidence interval. Coefficient: regression coefficient (slope) of the instrumental variable analysis with cluster-robust maximum likelihood estimation, including schools (clusters) as random effects, and adjusted for the baseline levels of student mental health (i.e., risk for depression, social-emotional-behavioural functioning, well-being), cohort, school size, school sex, and country. p: p-value associated with the slope.

Supplementary Table S16: Instrumental Variable Analysis of outcomes at post-intervention, with trial arm as an instrument for quality after being dichotomised

Outcome/Implementation variables	N (K)	coefficient	95% CI	p
CESD				
Quality: competent or more	6,440 (73)	0.53	-0.28 to 1.34	0.197
SDQ				
Quality: competent or more	6,334 (73)	0.33	-0.15 to 0.80	0.178
WEMWBS				
Quality: competent or more	6,415 (73)	-0.59	-1.31 to 0.14	0.115

Quality has been dichotomised as incompetent/beginner/advanced beginner vs competent/proficient/advanced.

N = number of students in analysis. K = number of clusters (schools) in analysis.

Supplementary Table S17: Raw descriptive data for the mediating factors by trial arm status and overall

Student (individual) characteristics	Intervention	Control	Total
Pre-intervention	N = 3,993	N = 3,931	N = 7,924
Mindfulness skills (CAMM), mean (SD)	27.62 (7.94)	27.49 (7.80)	27.56 (7.87)
Post-intervention	N = 3,703	N = 3,769	N = 7,472
Mindfulness skills (CAMM), mean (SD)	25.98 (8.30)	26.74 (8.26)	26.36 (8.29)
Pre-intervention	N = 3,259	N = 3,380	N = 6,639
Executive function (BRIEF2), mean (SD)	83.97 (21.09)	83.35 (20.43)	83.66 (20.76)
Post-intervention	N = 3,115	N = 3,426	N = 6,541
Executive function (BRIEF2), mean (SD)	85.68 (22.18)	84.95 (21.88)	85.30 (22.03)
Pre-Post (delta scores)	N = 3,571	N = 3,630	N = 7,201
Mindfulness skills (CAMM), mean (SD)	-1.70 (7.03)	-0.72 (6.82)	-1.21 (6.94)
Pre-Post (delta scores)	N = 2,658	N = 2,988	N = 5,646
Executive function (BRIEF2), mean (SD)	2.33 (15.86)	1.67 (15.19)	1.98 (15.51)

CAMM: Child and Adolescent Mindfulness Measure. BRIEF2: Behavior Rating Inventory of Executive Function 2.

References for the Supplements

1. Segal ZV, Williams JMG, Teasdale JD. Mindfulness-Based Cognitive Therapy For Depression. New York: Guildford Press 2013.
2. Mindfulness in Schools Project. (2016). How to teach .b. Teachers Notes. Curriculum available from: http://mindfulnessinschools.org/.
3. Huppert FA, Johnson DM. A controlled trial of mindfulness training in schools: The importance of practice for an impact on well-being. *Journal of Positive Psychology*2010;5:264-274 doi: 10.1080/17439761003794148 [published Online First: 3 August 2010].
4. Kuyken W, Weare K, Ukoumunne OC, et al. Effectiveness of the Mindfulness in Schools Programme: non-randomised controlled feasibility study. *British Journal of Psychiatry*2013;203:126-131 doi:10.1192/Bjp.Bp.113.126649 [published Online First: 2 January 2018].
5. Tudor K, Raja A, Baer R et al. Universal mindfulness training in schools for adolescents: A scoping review and conceptual model of moderators, mediators and implementation factors. *Prevention Science*2021; doi: 10.1007/s11121-022-01361-9 [published Online First: 10 March 2022].
6. Kuyken W, Bernard P, Baer R. Mindfulness-based Cognitive Therapy for Life Teacher's Guide. *Manuscript in preparation*: Guilford Press; 2021.
7. Crane C, Ganguli P, Ball S, et al. Training school teachers to deliver a mindfulness program: exploring scalability, acceptability, effectiveness, and cost-effectiveness. *Global Advances In Health And Medicine*2020;9:1-15 doi:10.1177/2164956120964738 [published Online First: 15 December 2020].
8. Montero-Marin J, Taylor L, Crane C et al. Teachers finding peace in a frantic world': An experimental study of self-taught and instructor-led mindfulness program formats on acceptability, effectiveness and mechanisms. *Journal of Educational Psychology*2021;113(8):1689-1708. doi: 10.1037/edu0000542. [published Online First: 18 October 2021].
9. Department of Education. Personal, social, health and economic (PSHE) education: A mapping study of the prevalent models of delivery and their effectiveness. London; 2010. Contract No.: DFE-RR080.
10. Kuyken W, Nuthall E, Byford S et al. The effectiveness and cost-effectiveness of a mindfulness training programme in schools compared with normal school provision (MYRIAD): study protocol for a randomised controlled trial. *Trials*2017;18:194 doi:10.1186/s13063-017-1917-4 [published Online First: 26 April 2017].
11. Montero-Marin J, Nuthall E, Byford S, et al. Update to the effectiveness and cost-effectiveness of a mindfulness training programme in schools compared with normal school provision (MYRIAD): study protocol for a randomised controlled trial. *Trials*2021;22:254 doi:10.1186/s13063-021-05213-9 [published Online First: 7 April 2021]
12. Formby E, Coldwell M, Stiell B, et al. Personal, Social, Health and Economic (PSHE) Education: A mapping study of the prevalent models of delivery and their effectiveness. London: Department for Education; 2010.
13. Weare K, Nind M. Mental health promotion and problem prevention in schools: what does the evidence say? *Health Promotion International*2010;25:29-69 doi: 10.1093/heapro/dar075 [published Online First: 1 December 2011].

14. Greenberg MT, Domitrovich C, Bumbarger B. Preventing mental disorders in school aged children. A review of the effectiveness of prevention programmes. Prevention Research Center for the Promotion of Human Development, College of Health and Human Development Pennsylvania State University. 2010
15. Association of Alaska School Boards. School climate and connectedness survey [measurement instrument]. Juneau: Association of Alaska School Boards; 2015.
16. Radloff LS. The use of the center for epidemiologic studies depression scale in adolescents and young-adults. *Journal of Youth and Adolescence*1991;20:149-66 doi: 10.1007/BF01537606 [published Online First: April 1991].
17. Rushton JL, Forcier M, Schectman RM. Epidemiology of depressive symptoms in the National Longitudinal Study of Adolescent Health. *Journal of the American Academy of Child and Adolescent Psychiatry*2002;41:199–205 doi:10.1097/00004583-200202000-00014 [published Online First: February 2002].
18. Goodman R. Psychometric properties of the Strengths and Difficulties Questionnaire. *J Am Acad Child Adolesc Psychiatry*2001;40:1337–45 doi: 10.1097/00004583-200111000-00015 [published Online First: November 2001].
19. Goodman R, Renfrew D, Mullick M. Predicting type of psychiatric disorder from Strengths and Difficulties Questionnaire (SDQ) scores in child mental health clinics in London and Dhaka. *Eur Child Adolesc Psychiatry*2000;9(2):129-34 doi:10.1007/s007870050008 [published Online First: June 2000].
20. Tennant R, Hiller L, Fishwick R et al. The Warwick-Edinburgh mental well-being scale (WEMWBS): development and UK validation. *Health Qual Life Outcomes*2007;5:63 doi:10.1186/1477-7525-5-63 [published Online First: 27 November 2007].
21. Clarke A, Friede T, Putz R, et al. Warwick-Edinburgh Mental Well-being Scale (WEMWBS): validated for teenage school students in England and Scotland. A mixed methods assessment. *BMC public health*2011;11:487 doi: 10.1186/1471-2458-11-487 [published Online First: 21 June 2011].
22. Crane RS, Koerbel L, Sansom S, et al. Assessing mindfulness-based teaching competence: good practice guidance. *Global advances in Health and Medicine*2020;9 doi:10.1177/2164956120973627 [published Online First: 9 November 2020].
23. Crane RS, Eames C, Kuyken W et al. Development and validation of the Mindfulness-Based Interventions - Teaching Assessment Criteria (MBI:TAC). *Assessment*2013;20:681-688 doi:10.1177/1073191113490790 [published Online First: 21 June 2013].
24. Crane RS, Kuyken W, Williams JMG. Competence in teaching mindfulness-based courses: Concepts, development and assessment. *Mindfulness*2012;3:8 doi:10.1007/s12671-011-0073-2 [published Online First 22 September 2011]
25. Panayiotou M, Humphrey N, Hennessey A. Implementation matters: Using complier average causal effect estimation to determine the impact of the Promoting Alternative Thinking Strategies (PATHS) curriculum on children's quality of life. *Journal of Educational Psychology*2020;112(2):236-253 doi:10.1037/edu0000360 [published Online First: 8 April 2019].
26. Greco LA, Baer RA, Smith G. Assessing mindfulness in children and adolescents: Development and validation of the child and adolescent mindfulness measure (CAMM). *Psychological Assessment*2011;23:606-614 doi:10.1037/a0022819 [published Online First: September 2011].

27. de Bruin EI, Zijlstra BJH, Bögels SM. The meaning of mindfulness in children and adolescents: further validation of the child and adolescent mindfulness measure (CAMM) in two independent samples from The Netherlands. *Mindfulness*2013;5(4):422-30 doi: 10.1007/s12671-013-0196-8 [published Online First: 6 February 2013].
28. Kuby AK, McLean N, Allen, K. Validation of the Child and Adolescent Mindfulness Measure (CAMM) with non-clinical adolescents. *Mindfulness*2015;6:1448–1455 doi:10.1007/s12671-015-0418-3 [published Online First: 22 May 2015].
29. Gioia GA, Isquith PK, Guy SC et al. Behavior Rating Inventory of Executive Function®, Second Edition (BRIEF®2). Lutz, FL: PAR Inc.2015
30. Ford T, Degli Esposti M, Crane C, et al. The role of schools in early adolescents' mental health: findings from the MYRIAD study. *Journal of the American Academy of Child and Adolescent Psychiatry*2021;60:1467-1478 doi: 10.1016/j.jaac.2021.02.016 [published Online First: December 2021].
31. Nylund KL, Asparouhov T, Muthén BO. Deciding on the number of classes in latent class analysis and growth mixture modeling: a monte carlo simulation study. *Structural Equation Modeling: A Multidisciplinary Journal*2007;14:535-569 doi: 10.1080/10705510701575396 [published Online First: 5 December 2007].
32. Bakk Z, Vermunt JK. Robustness of stepwise latent class modeling with continuous distal outcomes. *Structural Equation Modeling: A Multidisciplinary Journal*2016;23:20-31 doi: 10.1080/10705511.2014.955104 [published Online First: 15 May 2015].
